# Spatiotemporal Regulation of Cell Fate in Living Systems Using Photoactivatable Artificial DNA Membraneless Organelles

**DOI:** 10.1021/acscentsci.4c00380

**Published:** 2024-05-21

**Authors:** Lili Zhang, Mei Chen, Zhiqiang Wang, Minjuan Zhong, Hong Chen, Ting Li, Linlin Wang, Zhihui Zhao, Xiao-Bing Zhang, Guoliang Ke, Yanlan Liu, Weihong Tan

**Affiliations:** † Molecular Science and Biomedicine Laboratory (MBL), State Key Laboratory of Chemo/Biosensing and Chemometrics, College of Chemistry and Chemical Engineering, College of Biology, Aptamer Engineering Center of Hunan Province, Hunan University, Changsha, Hunan 410082, China; ‡ The Key Laboratory of Zhejiang Province for Aptamers and Theranostics, Zhejiang Cancer Hospital, Hangzhou Institute of Medicine (HIM), Chinese Academy of Sciences, Hangzhou, Zhejiang 310022, China; § Institute of Molecular Medicine (IMM), Renji Hospital, Shanghai Jiao Tong University School of Medicine, and College of Chemistry and Chemical Engineering, Shanghai Jiao Tong University, Shanghai 200240, China; ⊥ Molecular Science and Biomedicine Laboratory (MBL), State Key Laboratory of Chemo/Biosensing and Chemometrics, College of Chemistry and Chemical Engineering, College of Materials Science and Engineering, Aptamer Engineering Center of Hunan Province, Hunan University, Changsha, Hunan 410082, China

## Abstract

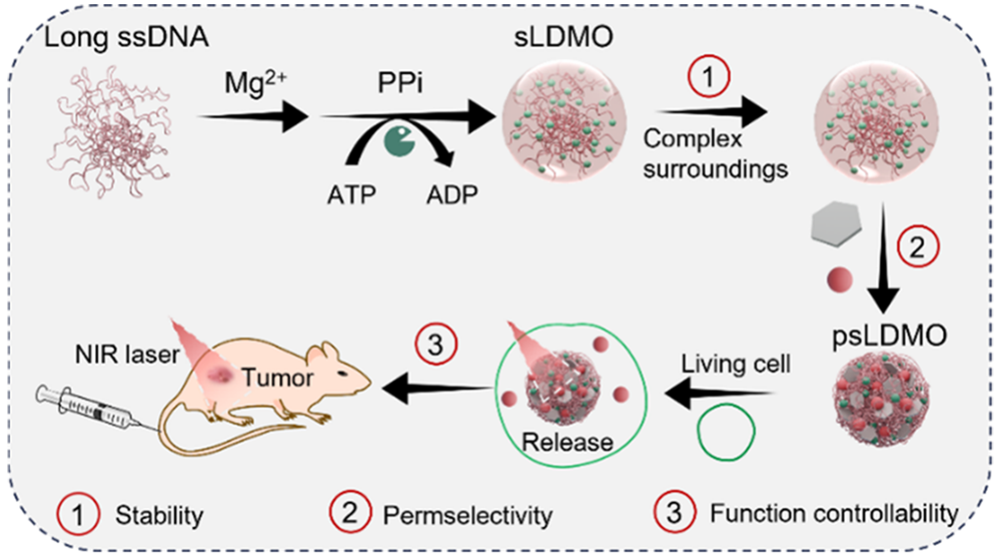

Coacervates formed by liquid–liquid phase separation emerge as important biomimetic models for studying the dynamic behaviors of membraneless organelles and synchronously motivating the creation of smart architectures with the regulation of cell fate. Despite continuous progress, it remains challenging to balance the trade-offs among structural stability, versatility, and molecular communication for regulation of cell fate and systemic investigation in a complex physiological system. Herein, we present a self-stabilizing and fastener-bound gain-of-function methodology to create a new type of synthetic DNA membraneless organelle (MO) with high stability and controlled bioactivity on the basis of DNA coacervates. Specifically, long single-strand DNA generated by rolling circle amplification (RCA) is selected as the scaffold that assembles into membraneless coacervates via phase separation. Intriguingly, the as-formed DNA MO can recruit RCA byproducts and other components to achieve self-stabilization, nanoscale condensation, and function encoding. As a proof of concept, photoactivatable DNA MO is constructed and successfully employed for time-dependent accumulation and spatiotemporal management of cancer in a mouse model. This study offers new, important insights into synthetic membraneless organelles for the basic understanding and manipulation of important life processes.

## Abstract 2

Coacervate membraneless organelles formed through liquid−liquid phase separation (LLPS) have been actively investigated as an emerging cargo delivery platform to harbor proteins, RNAs, and therapeutic molecules. However, a trade-off among structural stability, versatility, and molecular communication in physiological conditions hampers their application for systemic administration. Our study develops self-stabilizing and fastener-bound gain-of-function DNA membraneless organelles (MOs). Unlike traditional approaches to obtain functional MOs via complicated surface coating or hybridization, long single-strand DNA coacervates are used as scaffolds, enabling self-stabilization and function encoding by simply recruiting surrounding components during LLPS for systematic regulation of cell fate, without loss of communication properties.

## Introduction

Endogenous membraneless organelles (MOs) are spontaneously organized as biomolecular condensates via liquid–liquid phase separation (LLPS), having pivotal roles in multiple biological processes including gene transcription, protein modification, signal transduction, and cellular detoxification. For cellular detoxification processes, MOs can instantly isolate and remove the dangerous molecules from the intracellular surrounding milieu by activating stress-responsive pathways.^1−3^ They have been an underlying defense tool against threats.^4−6^ When stress disappears, MOs will depolymerize in time due to the lack of membrane structure.^7,8^ The dynamic nature of the MOs determines that they can directly orchestrate pathological events in a local cell or obliquely promote systemic effects of stress via paracrine and endocrine pathways.^9,10^ Nevertheless, the distant and indirect regulation may cause loss of information, resulting in a limited communication effect.^11,12^ Seriously, negative communication risks will be exposed by MOs when cells are under continuous external stimuli.^13−15^ However, the current research on MOs is still in its infancy, so it is difficult to accurately control the MO’s structure for spatiotemporally switching on and off its regulation toward cell fate in a complex physiological environment.

**Figure 1:**
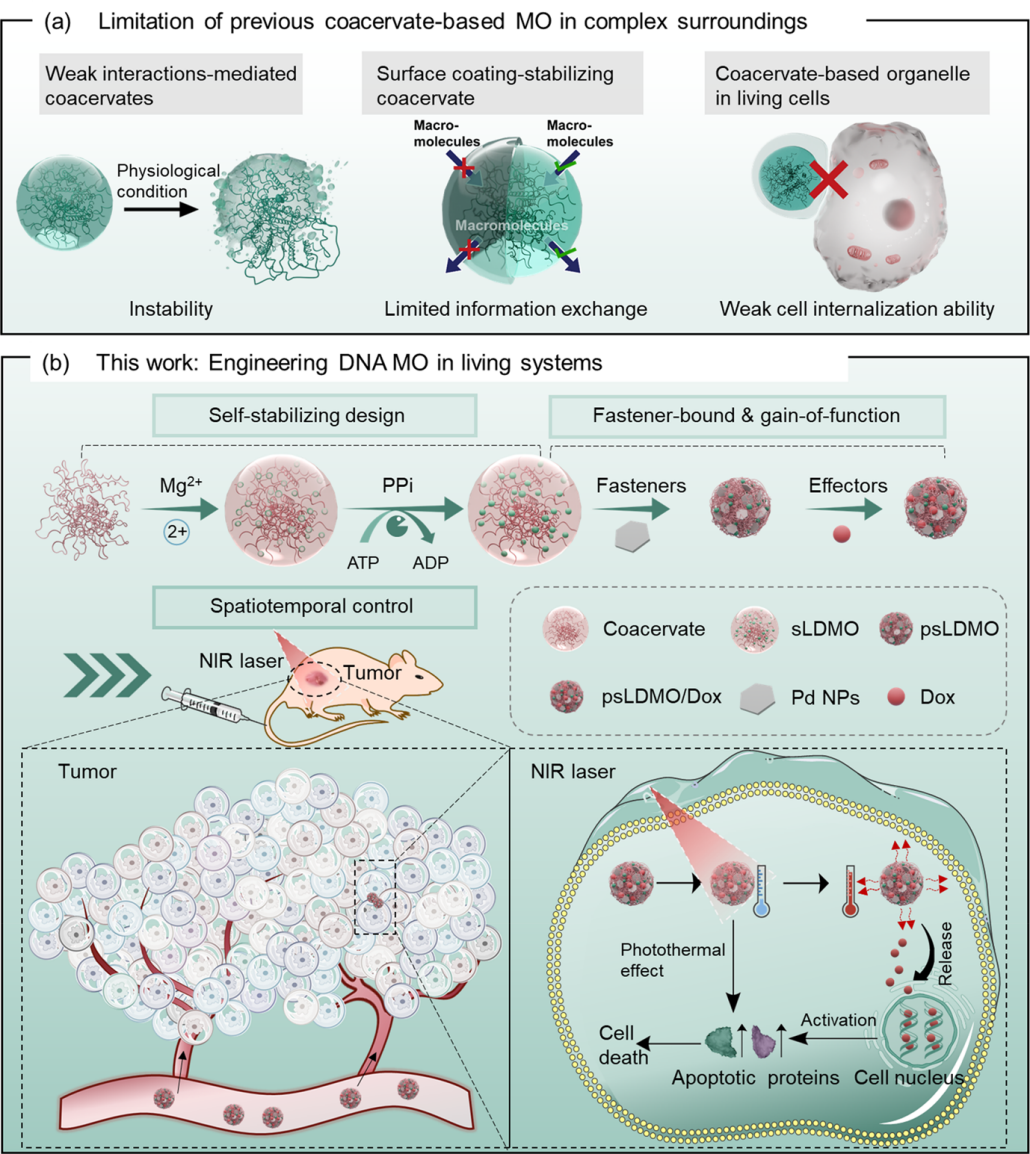
Schematic illustration of the LLPS-driven construction of self-stabilizing and fastener-bound photoactivatable DNA MOs and their use for spatiotemporal regulation of the cell fate of cancer cells *in vivo*. (a) Previous coacervates are usually constructed by LLPS via noncovalent interaction, such as electrostatic interaction, thereby suffering from the limitations of metastable structures, poor permeability to macromolecules, and uncontrollable function manipulation. (b) Long, single-stranded DNAs are first generated by rolling circle amplification (RCA). They, in turn, recruit RCA byproducts (pyrophosphate ions, PPi) for self-stabilization in the presence of Mg^2+^ to form self-stabilizing DNA membraneless coacervates under LLPS. Similar to natural inflammasomes, the as-formed coacervates can be further condensed and functionalized after bonding with other species. For the purpose of spatiotemporal function manipulation, Pd NPs are used as the nanofastener, and Dox is employed as the effector molecules to produce photoactivable DNA MOs. Upon systemic administration, such stable DNA MOs demonstrate high tumor accumulation, causing programmable tumor cell death and reshaping the tumor microenvironment via synergistic photothermal effects and effector-mediated chemical interference upon NIR laser irradiation.

Bottom-up re-creation of membraneless organelles by using coacervates has been emerging as a powerful means to address this dilemma owing to its ability to motivate the engineering of smart architectures with organelle-like properties for the direct regulation of cell fate in a controlled manner.^16−19^ To date, numerous coacervates have been constructed as synthetic membraneless organelles harboring cargoes, displaying great promise as an underlying defense tool to orchestrate pathological events, such as pathological regulation,^20,21^ homeostasis balance,^22^ and intracellular pathway activation,^4^ because of enhanced cellular internalization mediated by electrostatic interactions,^22,23^ hydrophobic interactions,^24^ or a lipid-raft-mediated transmembrane pathway.^19,25^ Moreover, surface coating effectively improves the stability and intervention effect of diseases in complex physiological environments.^26^ However, these coacervates suffer from some new weaknesses, including limited MO dynamics,^27^ uncontrollable loading and release of functional cargoes,^28^ and poor cellular internalization as a result of the microsized structures (Figure 1a).^29^ In addition, to the best of our knowledge, there are few coacervate MOs to spatiotemporally control cell activity in living systems. This calls for the development of innovative strategies capable of spatial condensation, positive accumulation, and controllable release of signal molecules in order to promote the expansion of lifelike functions in artificial entities for applications in living systems.

Aiming at achieving this goal, we noticed inflammasomes, a type of LLPS-driven natural multiprotein complex involved in innate immune defense. In response to harmful signals, inflammasomes serve as “microreactors” to recruit downstream adaptor proteins, which can both condense the coacervate structure and concurrently promote on–off activation of effectors (e.g., caspases), causing programmed cell death and reshaping the microenvironment of adjacent tissues.^6,30^ Inspired by such a bioprocess, here we present a self-stabilizing and fastener-bound gain-of-function methodology to create synthetic membraneless organelles with high stability and spatiotemporally controlled bioactivity. To design a platform for such organelles, we take advantage of the key findings from previous studies which reported how long, single-stranded DNA generated by rolling circle amplification (RCA) could assemble into microscale coacervates via a phase separation process.^31^ Intriguingly, the as-formed programmable DNA coacervates could recruit RCA byproducts and other components to achieve self-stabilization and function encoding, analogous to the formation dynamics of natural inflammasomes (Figure 1b).^32^ In order to gain controllable activity decoding, Pd nanoparticles are introduced as a photosensitive fastener similar to the adaptor of inflammasomes, and doxorubicin (Dox) is used as a proof-of-concept effector, yielding the final artificial DNA MOs. Benefiting from self-stabilization and fastener-bound condensation effects, the engineered organelle demonstrates excellent tolerance to complex physiological environments. More impressively, the photosensitive fastener enables NIR-responsive *in situ* activation of MOs and release of effector molecules, thus orchestrating downstream cell apoptotic/pyroptotic signaling of cancer cells in a spatiotemporally controlled manner. This artificial organelle has been successfully employed for time-dependent accumulation and spatiotemporal management of cancer in a xenograft mouse model. Owing to flexible function integration, this design promises to offer a versatile scaffold to create various evolutionary entities for an in-depth understanding of life source, operation, and evolution. This artificial membraneless organelle also bridges the gap between life science and materials science to facilitate the on-demand delineation and regulation of biological functions.

## Results and Discussion

### Phase Separation and Self-Stabilization of Long, Single-Stranded DNA

Disordered biomacromolecules, such as proteins and nucleic acids, can spontaneously transform into a state of low entropy and high order, which, under certain circumstances, is a thermodynamically favored process.^33,34^ LLPS would occur when the interactions between biomacromolecules exceed the repulsion and mixing entropy of the entire system, a mechanism governing the formation of traditional coacervate-based synthetic membraneless organelles.^34,35^ However, the as-formed coacervates are droplets in a nonequilibrium state and often undergo rapid rupture in living organisms because the interactions in coacervates are too weak to counteract the repulsion and mixing entropy of the complex physiological system.^36^

**Figure 2:**
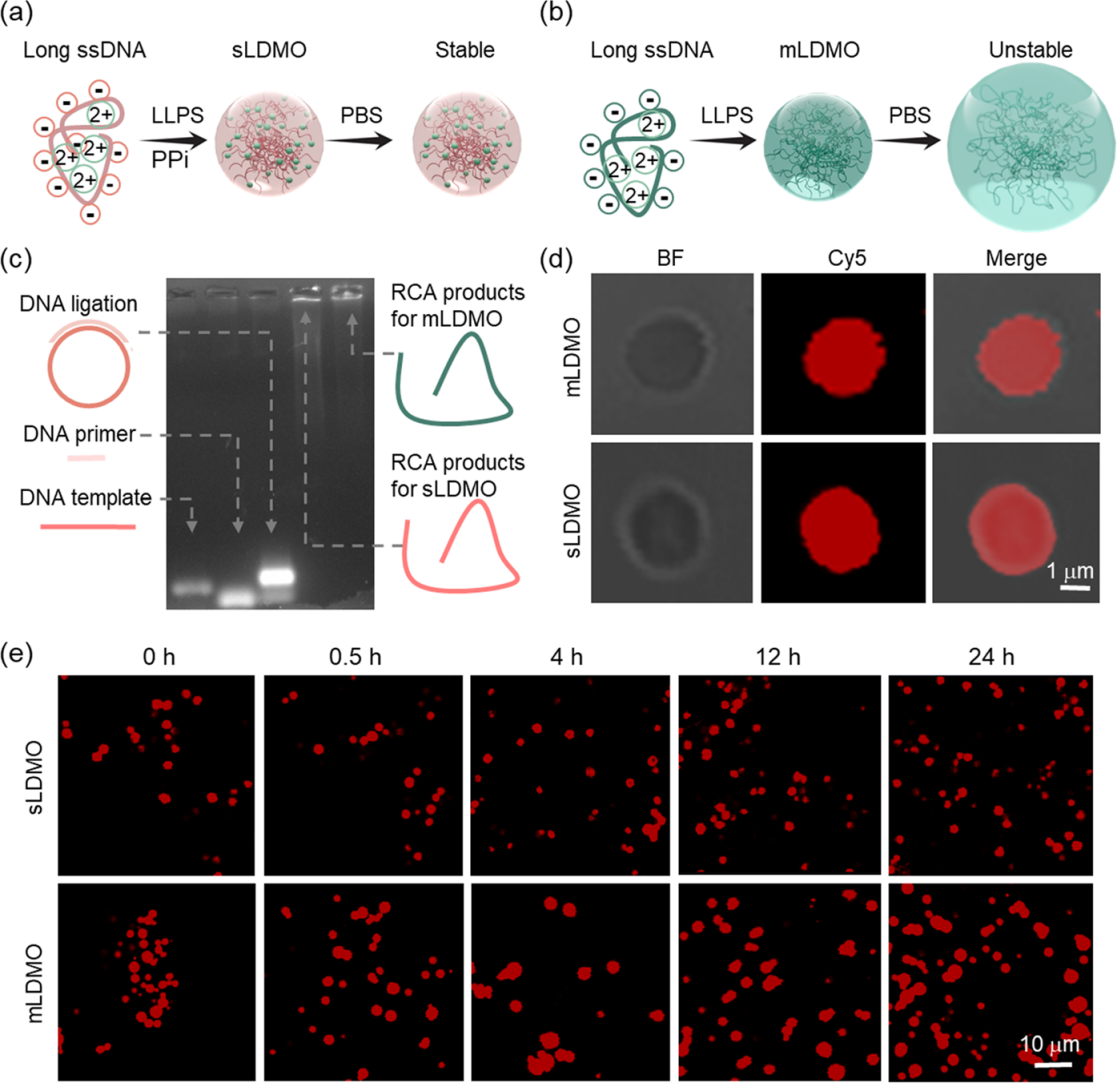
Construction and stability studies of artificial, long ssDNA membraneless organelles. Schematic illustration of the formation and behaviors of stable DNA MOs (sLDMOs) (a) and metastable DNA MOs (mLDMOs) (b) in PBS buffer, respectively. (c) Gel electrophoresis analysis of the formation of long ssDNA during RCA. (d) Confocal imaging of the as-formed metastable DNA MOs (mLDMOs) and the stable DNA MOs (sLDMOs) loaded with Cy5. (e) Time-dependent confocal imaging of Cy5-loaded sLDMOs or mLDMOs in a PBS buffer.

To overcome this limitation, we first selected DNA as the building unit, because of its programmable and assembly features. Specifically, long, single-stranded DNA (ssDNA) was first built by polymerizing circular dsDNA in the presence of DNA polymerase through an RCA-mediated ATP-consuming process (Figure S1 and Table S1). As time increased, the as-formed long ssDNA could translate into microscale droplet-like coacervates in the presence of magnesium (Mg^2+^) ions driven by LLPS. Of note, pyrophosphate ions (PPi), as the byproduct of RCA, could recruit more Mg^2+^ ions for *in situ* mineralization, thus accelerating phase separation owing to entropy-driven effects and stabilization of the DNA coacervates (Figure 2a) (hereinafter denoted as self-stabilizing, long ssDNA membraneless organelles (sLDMOs)). Gel electrophoresis analysis was carried out to prove this process. As a reference, mineralization-free metastable DNA MOs (mLDMOs) were also prepared by excluding PPi with pyrophosphatase (Figure 2b). Compared to the template or primer DNA, the polymerization products obtained with or without *in situ* mineralization moved more slowly than either the ssDNA (DNA template/primer) or DNA ligation and were largely trapped in the channel (Figure 2c), suggesting the successful amplification of short ssDNA and formation of long ssDNA via RCA for later assembly of mLDMOs and sLDMOs. Furthermore, the appearance of scattering in ultraviolet–visible (UV–vis) spectra and fluorescence imaging provided further evidence of the phase separation of long ssDNA (Figures S2, S3 and Figure 2d).

To demonstrate the impact of the self-stabilizing process on DNA MOs, a dynamic evaluation of sLDMO and mLDMO stability was performed after hybridization with Cy5-modified nucleotides. As shown in Figure 2e and Figure S4, mLDMOs became gradually swollen in PBS over time, primarily from permeation with the surroundings. In contrast, the morphology and size of sLDMOs were well maintained during the same period of time, suggesting that self-stabilization could remarkably enhance the resistance of DNA MOs toward environmental stress.

### Fastener-Bound Condensation and Photoactivation of DNA MOs

A key characteristic of natural membraneless organelles is their ability to provide spatially confined reaction compartments for intracellular/intercellular communications.^37−40^ On the other hand, strategic innovation enabling remote and spatiotemporal manipulation can promote the complexity of functionality and allow on-demand activation of bioactive components.^20^ To imitate and achieve spatiotemporal control over such a biological process, near-infrared (NIR)-absorbing Pd nanoparticles (NPs) with an average diameter of ∼13 nm were integrated into sLDMOs as the photoreactive fastener (Figure S5) through multivalent coordination interactions between DNA MOs and PVP on the Pd surface,^41^ yielding photoactivatable coacervates (psLDMOs). Pd NPs are well-known as one of the most effective NIR photosensitizers able to convert NIR light into heat,^19^ and as such, they are expected to trigger thermal expansion of DNA MOs for spatiotemporally controlled release of effectors. Gel electrophoresis demonstrated the absence of a bright band in the entire gel upon loading of the photoreactive fastener-bound psLDMOs. Instead, the channel became much darker, as compared to sLDMOs (Figure S6a). On the contrary, the mixing of Pd NPs with primer or template DNAs had no obvious effect on DNA migration and brightness of bands, albeit accompanied by the presence of darkened channels (Figure S6b). We reasoned that the larger amount of coordination sites in DNA MOs relative to short primer or template DNA enabled strong and high-capacity binding with Pd NPs, leading in turn to “band quenching” by the dark Pd NPs. Based on inductively coupled plasma optical emission spectroscopy (ICP-OES) and energy dispersive X-ray analysis (EDAX) examination (Table S2 and Figure S7), psLDMOs were determined to contain 59.8% Pd NPs by weight, providing additional support of successful and strong recruitment of sLDMOs toward Pd-based photosensitive nanofasteners.

In natural inflammasomes, it has been suggested that recruitment of adaptors can lead to decay of liquid-like performance owing to the fastening effect of adaptors via multivalent interactions.^6,42^ Similarly, we found that a low concentration of photoreactive fasteners did have a negligible influence on the morphology of DNA MOs (Figure S8). When the fastener concentration continued to increase, microscale DNA MOs were condensed into irregular spherical nanostructures with an average size of 100 nm (Figure S9). However, such fastener-bound coacervate condensation would benefit their applications in living systems since micron-scale structures tend to be rapidly cleared from circulation.^43^

To gain more insight into fastener-bound coacervate condensation, Pd NPs were introduced during RCA, and the follow-up LLPS process for forming sLDMOs was monitored. Results showed that the Pd-based fastener could condense the as-formed large sLDMOs into smaller ones (Figure S10), though not into nanoscale. We reasoned that the strong and multivalent interactions between fastener and long ssDNA could facilitate effective phase separation and subsequent micrometer-to-nanometer condensation of DNA MOs via RCA in a time-dependent manner. Of note, the fastener-triggered condensation coacervate strategy was also amenable to metastable mLDMOs, as evidenced by the remarkably reduced size (Figure S11). However, when PPi was digested during LLPS, the following Pd binding could not stop mLDMOs from swelling under physiological conditions (Figures S12–S14). Besides, fluorescence recovery after photobleaching (FRAP) analysis was performed with FAM-labeled psLDMOs. Results showed that the engineered psLDMOs exhibited a solid-like feature, as evidenced by the uncovered green fluorescence (Figure S15). Taken together, fastener-bound condensation, along with the aforementioned self-stabilizing effect, contributed to the successful construction of a stable and condensed DNA MOs.

**Figure 3:**
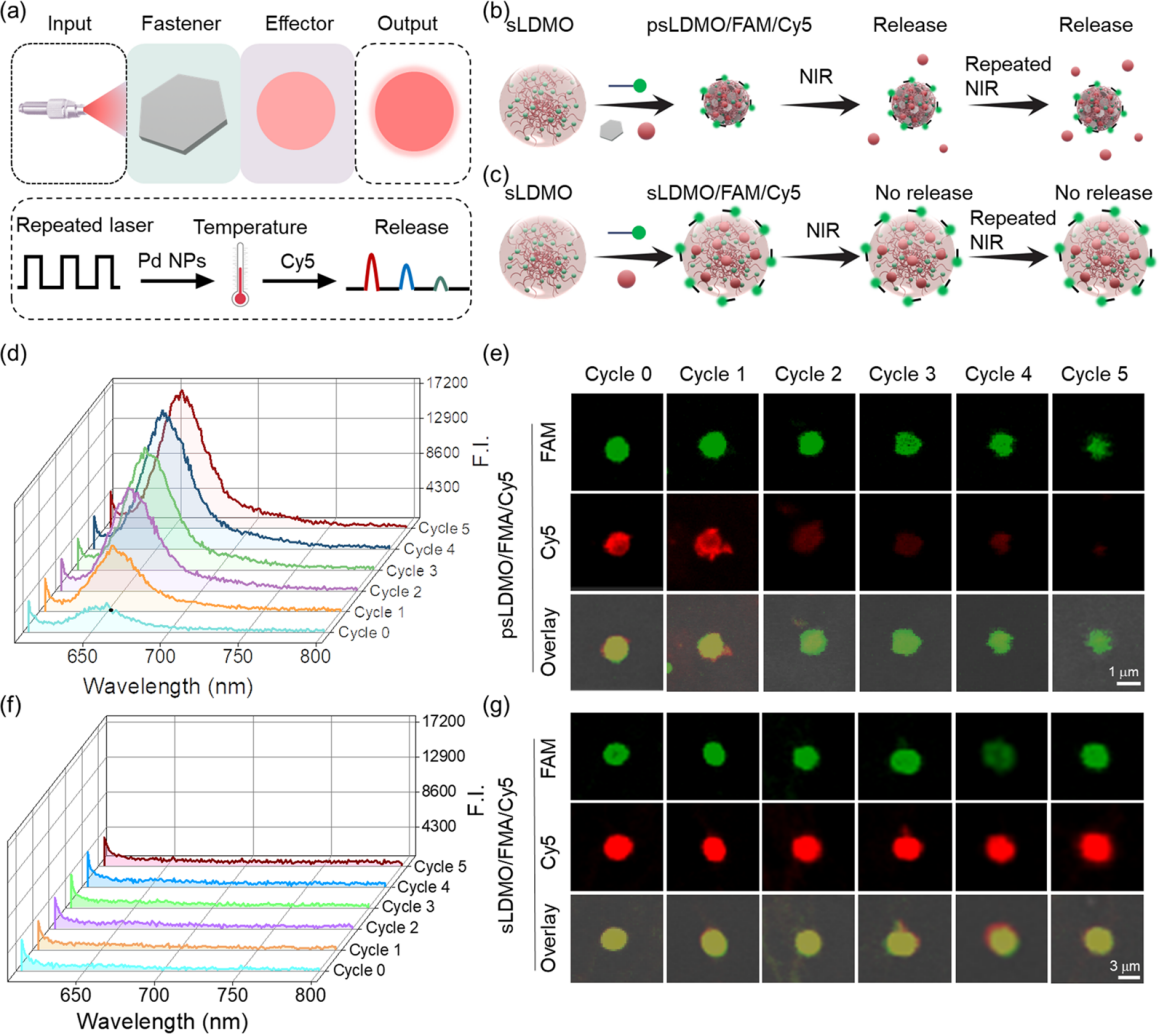
Spatiotemporal release of effectors in the model DNA organelles. (a) Schematic illustration of the structure and photoresponsivity of the artificial DNA organelle with Pd NPs and Cy5 as the fastener and effector, respectively. Schematic illustration of the light-responsive release of the loaded Cy5 from (b) photoreactive fastener-bound psLDMOs or (c) photoreactive fastener-free sLDMOs, in which FAM was labeled on DNA and hybridized into the structure. Fluorescence spectra (d) and imaging (e) of Cy5 release from FAM-labeled psLDMOs during five cycles of laser irradiation (808 nm, 10 W/cm^2^, 1 s). Fluorescence spectra (f) and imaging (g) of Cy5 release from FAM-labeled sLDMOs during five cycles of laser irradiation (808 nm, 10 W/cm^2^, 1 s).

Next, the photoresponsivity of the engineered photoactivatable coacervates and the release dynamics of effectors were studied (Figure 3a). For better observation, a large psLDMO with an average size of 1 μm obtained after 60 h RCA was used as a proof-of-concept illustration. Specifically, a psLDMO was conjugated with FAM and subsequently loaded with Cy5 as the model effector (Figure 3b). A time-dependent Cy5 release profile was recorded upon irradiation with an 808 nm laser. Meanwhile, the photoresponsivity of the sLDMO was compared (Figure 3c). After 1 s of irradiation at 10 W/cm^2^, Cy5 was rapidly released from the psLDMO and completed after 5 cycles of irradiation (Figure 3d). Furthermore, confocal imaging displayed the gradual loss of red fluorescence in psLDMOs, yet the green FAM fluorescence was slightly attenuated (Figure 3e). For nonresponsive sLDMOs, on the contrary, no significant Cy5 fluorescence was detected in the supernatant, and the red fluorescence in sLDMOs was almost unchanged during the five cycles of irradiation (Figure 3f, g, and Figure S16). Collectively, these findings strongly supported the structural stability and controlled activation of the photoactivatable DNA MO, which are expected to act as promising synthetic membraneless organelles for spatiotemporal regulation of applications in complex living systems.

### Spatiotemporal Regulation of Programmed Cell Death in Tumor Cells

**Figure 4:**
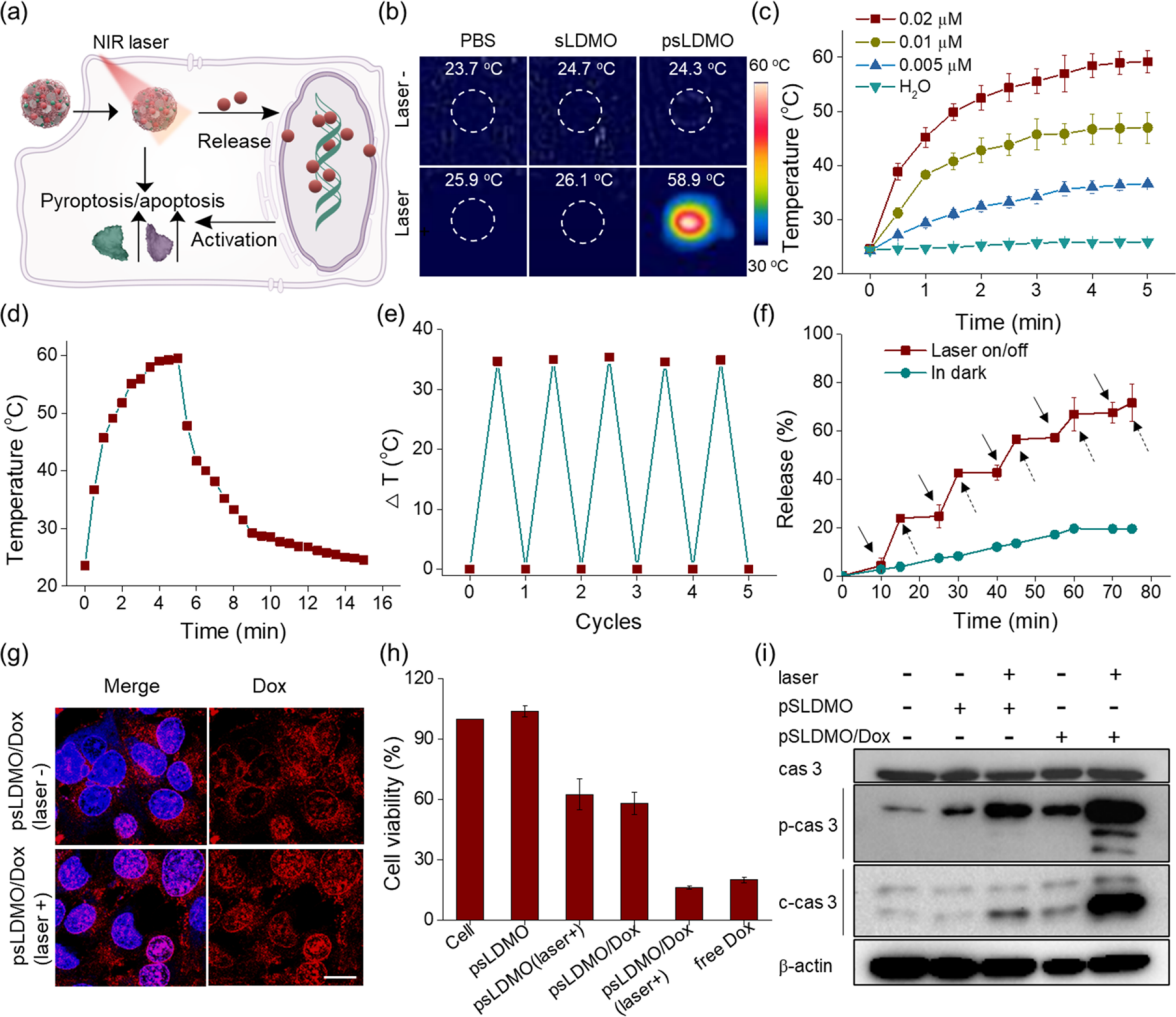
*In vitro* spatiotemporally controlled antitumor studies. (a) Schematic illustration of spatiotemporally controlled regulation of 4T1 cells with psLDMO/Dox. (b) IR thermal images of 4T1 cells after treatment with photoreactive fastener-free sLDMOs or photoreactive fastener-bound psLDMOs and in the absence vs presence of laser irradiation (808 nm, 2 W/cm^2^ for 5 min). (c) Time-dependent temperature changes in psLDMO aqueous solutions at different concentrations under laser irradiation (808 nm, 2 W/cm^2^). (d) Heating/cooling profile of the psLDMO solution (0.02 μM) under 808 nm of irradiation at 2 W/cm^2^. (e) Temperature changes of a psLDMO aqueous solution (0.02 μM) after five cycles of repeated on–off laser irradiation (808 nm, 2 W/cm^2^ for 5 min). (f) Dox release curves of psLDMOs/Dox (0.02 μM) with vs without on–off laser irradiation. Solid arrows indicate that the laser was switched on, and the dotted arrows indicate that the laser was switched off. (g) Confocal imaging of psLDMO/Dox-incubated 4T1 cells with vs without 5 min laser irradiation. (h) Cell viability of 4T1 cells after treatment with psLDMOs or psLDMOs/Dox under NIR light or in dark. (i) Western blot analysis of the expression of caspase 3, procaspase 3, and cleaved caspase 3 in 4T1 cells treated with psLDMOs or psLDMOs/Dox under NIR light or in the dark.

After having established photoresponsive properties of the engineered DNA MO, we proceeded to investigate their feasibility for spatiotemporal manipulation of important biological processes in live cells (Figure 4a). To achieve this purpose, we turned our attention to natural inflammasomes. As the sensor in innate immune defense, inflammasomes are capable of recruiting and regulating the activation of effectors, particularly inflammatory caspase (caspase 1, caspase 11, caspase 3) in response to infectious microbes, eventually initiating programmed cell death via pyroptosis and/or apoptosis.^44−46^ Inspired by this process, Dox was chosen as the caspase-activated effector and loaded in smaller psLDMOs obtained after 24 h RCA (Figures S17, S18) to construct functional nanoscale membraneless organelles for modulating programmed cell death of tumor cells in living systems.^47,48^ Impressively, the photoresponsive psLDMOs could efficiently recruit Dox upon mixing as a result of electrostatic and van der Waals interactions, with a high loading capacity of 68.6% (Figure S19). Moreover, Dox loading did not have any adverse influence on the photothermal conversion capability of psLDMOs (Figure 4b-d and Figure S20, S21). Upon irradiation with the 808 nm laser at 2 W/cm^2^, the engineered psLDMOs/Dox exhibited a dose- and time-dependent increase in the solution temperature. At a concentration of 0.02 μM, the solution temperature was increased to 60 °C without obvious decay in photothermal conversion efficacy after five on/off irradiation cycles (Figure 4e). In addition, the light-triggered unlocking of Dox was examined. In the dark, fast and sustained release of Dox was detected upon laser irradiation with 79% of release ratio after five on/off irradiation cycles (Figure 4f), whereas only a slight portion of weakly attached free Dox was dissociated from psLDMOs/Dox within the same time duration (20%) in the absence of irradiation, clearly indicating the controlled activation of psLDMOs/Dox.

Given the triggerable activation property of psLDMOs/Dox, we next assessed its feasibility for spatiotemporal activation and regulation of programmed cell death in tumor cells. To do this, 4T1 cells, a murine mammary carcinoma cell line, were incubated with psLDMOs/Dox for a specific time, followed by treatment with or without laser irradiation. Confocal imaging showed a gradually lighted red fluorescence in the cytoplasm, suggesting time-dependent internalization of psLDMOs/Dox through the endocytosis mechanism (Figure 4g, Figure S22–S23, and Table S3). Most Dox molecules were still trapped within psLDMOs/Dox under dark. In contrast, Dox molecules were effectively released from psLDMOs/Dox in 4T1 cells after exposure to irradiation, as evidenced by the high fluorescence overlap between Dox (red) and Hoechst (blue) channels. Next, the effect of psLDMOs/Dox on cell viability was determined (Figure 4h). Compared with cells alone, Dox-free psLDMOs elicited no significant cell death. Meanwhile, more than 59% of cells remained alive after incubation with psLDMOs/Dox in the dark. In sharp contrast, nearly 80% of cells were killed in cells treated psLDMOs/Dox along with laser irradiation, as a consequence of light-controlled activation and fast release of Dox in psLDMOs/Dox. To validate whether the antitumor effect of psLDMOs/Dox was dominated by programmed cell death mechanisms, the activation of caspase signaling pathways in these experimental groups was determined by Western blot analysis. Consistent with results from cell viability assay, cells treated with combined psLDMOs/Dox and laser demonstrated the highest expression of cleaved caspase 3 and pro-caspase 3 at the protein level among these tested groups (Figure 4i) in contrast to the negligible or weak activation of this programmed death protein in other groups.^49−51^

### *In Vivo* Light-Triggerable Programmed Cell Death in Tumor Models

Encouraged by the good *in vitro* results, we asked if such light-triggerable programmed cell death could be achieved *in vivo* by photoactivatable DNA MOs. Before *in vivo* light-triggerable programmed cell death, DLS analysis was performed, and the drug release profile of psLDMOs in 10% FBS solution was measured to investigate its physiological stability. Neither obvious size changes of psLDMOs under physiological conditions (Figure S13, S14, and S24) nor evident Dox leakage from psLDMOs/Dox was detected (Figure S25), suggesting the good structural stability of psLDMOs in serum solution. In addition, the pharmacokinetics and biodistribution of these photoactivatable DNA MOs were evaluated. Benefiting from nanoscale size and good structural stability, psLDMOs was found to exhibit prolonged circulation in the blood relative to free DNA after intravenous injection (Figure S26). Subsequently, 4T1 xenograft tumor models were established and injected with Cy5-labeled psLDMOs (psLDMOs/Cy5) through the tail vein for biodistribution studies. Time-dependent accumulation of psLDMOs in the tumor was found (Figure S27). Moreover, the level of psLDMOs in tumors was higher than that in other organs(Figure S28). To further demonstrate the biodistribution, the tumor-bearing mice were subjected to intravenous injection of Dox-loaded psLDMOs (psLDMOs/Dox), followed by fluorescence of Dox (Figure S29–S31). Consistent with the above results, the level of psLDMOs in the tumor was higher than that in other organs. Of note, the fluorescence of Dox in tumor could not be observed during whole-body fluorescence imaging because of the limited penetration depth of Dox’s fluorescence.

**Figure 5:**
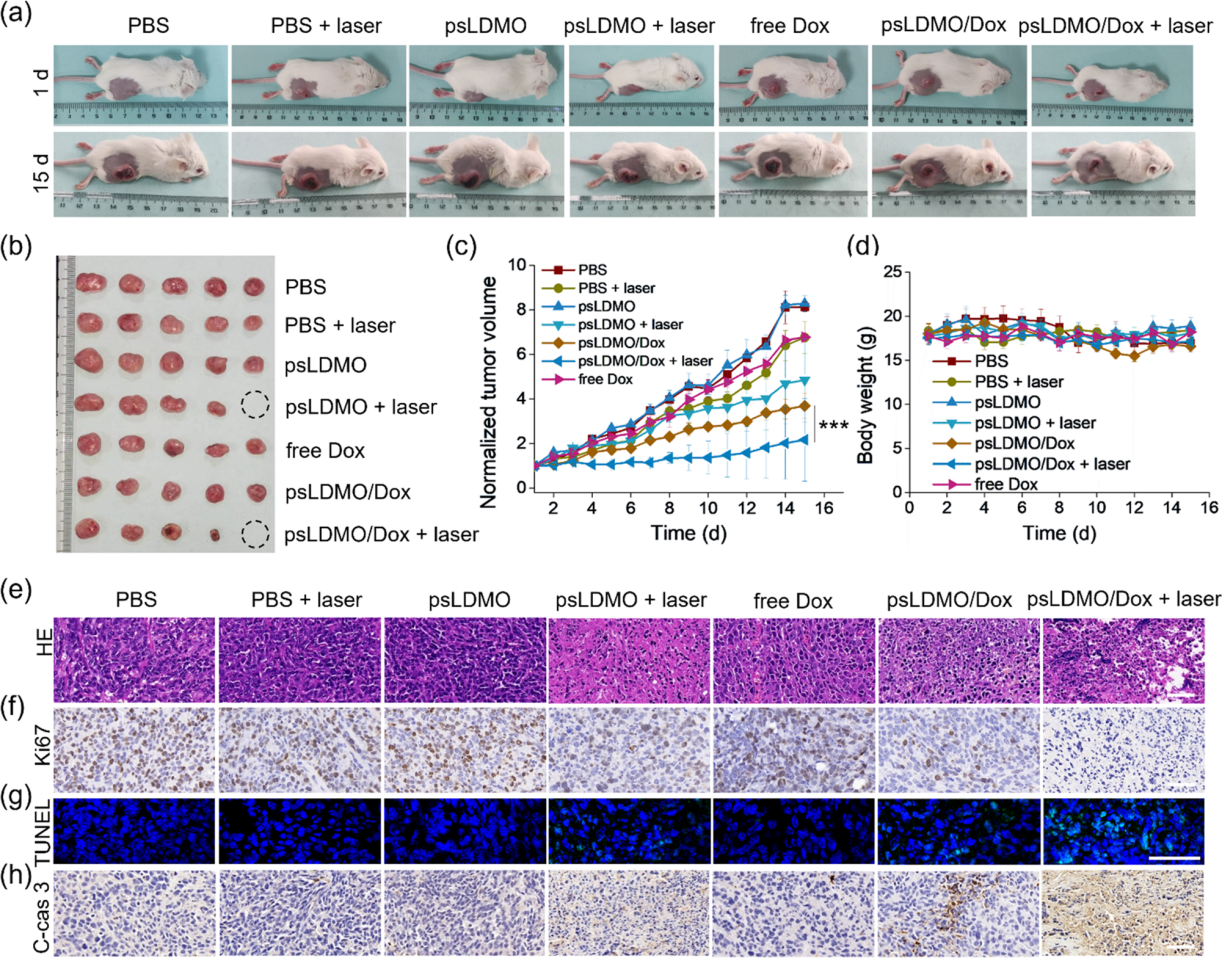
*In vivo* spatiotemporally controlled antitumor effects. (a) Representative digital pictures of 4T1 tumor-bearing BALB/c mice before and on day 15 after treatment with PBS, PBS + laser, psLDMOs, psLDMOs + laser, free Dox, psLDMOs/Dox, and psLDMOs/Dox + laser, respectively. (b) Digital picture of tumors collected from various groups on day 15 (*n* = 5). (c) Tumor volume growth curves of these groups during a 15-day treatment period. Data are shown as mean ± SD (*n* = 5). ***P < 0.001. (d) Body weight changes of these groups during treatment. Representative histological staining images of tumor tissues from different groups. H&E staining (e), immunohistochemical staining of Ki67 (f), TUNEL immunofluorescence (g), and immunohistochemical staining (h) of cleaved caspase 3. The scale bar is 50 μm.

Next, 4T1 xenograft tumor models were randomly divided into seven groups including (i) PBS, (ii) PBS + laser, (iii) psLDMOs, (iv) psLDMOs + laser, (v) psLDMOs/Dox, (vi) psLDMOs/Dox + laser, and (vii) free Dox. Similar to observations *in vitro*, the temperature of tumor tissue from mice treated with psLDMOs (iv) or psLDMOs/Dox (vi) was dramatically increased (Figure S32), stemming from the photothermal conversion capability of Pd-based fasteners. No obvious temperature increase was detected in other groups. After treatment, the tumor growth in these groups was monitored every day for 2 weeks (Figure 5a-d and Figure S33–S35). Evidently, the tumor in mice that received a laser or psLDMOs alone grew in a manner similar to that of the PBS control group. Treatment with psLDMOs/Dox or psLDMOs + laser-induced a delay in tumor growth owing to Dox- or laser-induced hyperthermia, respectively. Impressively, tumor growth in mice treated with psLDMOs/Dox accompanied by laser exposure was significantly suppressed, benefiting from the synergistic effect of light-triggered hyperthermia and adaptive unlocking of Dox. Moreover, the body weight of mice was comparable to that of the control group (Figure 5d). To further validate the potential of psLDMOs/Dox for spatiotemporal manipulation of cell fate, tumor tissues were harvested at the therapeutic end point, followed by histological examination. It was evident that only psLDMOs/Dox + laser treatment induced severe damage to tumor tissue, as displayed in the hematoxylin and eosin (H&E) staining images (Figure 5e). In parallel, immunofluorescence and immunohistochemical analyses also confirmed the positive TUNEL and cleaved caspase 3, yet negative Ki67, staining in the psLDMOs/Dox + laser treatment group (Figure 5f-h), suggesting the significant programmed cell death and growth inhibition of tumor cells. Next, the possible adverse effects of the engineered photoactivated DNA MOs were assessed. Neither tissue damage nor unwanted inflammation was observed in the slices from other organs. In parallel, blood biochemical indicators levels of alanine transaminase (ALT), aspertate aminotransferase (AST), total bilirubin (TBIL), albumin (ALB), direct bilirubin (DBIL), total bile acid (TBA), alkaline phosphatase (ALP), γ-glutamyltransferase (γ-GT), UREA, creatinine (CREA), and uric acid (UA) in serum were similar between the experimental group and control group (Figure S36–S38). While in-depth *in vivo* toxicity evaluations are still needed, these preliminary results indicated that the engineered psLDMOs could be a potential candidate as a synthetic membraneless organelle for functional manipulations in living organisms.

## Conclusions

In summary, we have presented a fastener-bound self-stabilizing and gain-of-function methodology to establish a platform for engineering synthetic DNA membraneless organelles with high stability and controllable bioactivity. The central principle of the idea is inspired from natural inflammasomes, a class of LLPS-driven multiprotein oligomers responsible for inflammatory modulation through the recruitment of adaptors and activation of downstream effectors. In this work, DNA MOs were assembled from long, single-stranded DNA via LLPS. Analogous to inflammasomes, DNA MOs could realize self-stabilization through recruitment of large amounts of Mg^2+^ and subsequent biomineralization with the byproduct during the generation of long, single-stranded DNA. Furthermore, the engineered DNA MOs could bind other types of fasteners conferring nanoscale condensation of DNA MOs endowed with designated functionalities. As a proof of concept, we have selected NIR-absorbing Pd nanoparticles as the nanofastener and the anticancer drug Dox as the model effector to construct photoactivable synthetic DNA membraneless organelles for spatiotemporal regulation of programmed cell death in living systems. In-depth studies demonstrate that the engineered photoactivable DNA MOs exhibited high structural stability, strong photothermal conversion capability, high-capacity binding with the fastener and effector, and light-triggerable unlocking behaviors, all in complex biological systems, contributing to efficient and spatiotemporally controlled regulation of tumor cell fate at both the cellular and animal levels. This work provides new insights into understanding and bottom-up re-creation of cellular processes into a synthetic system and may offer a balanced solution to achieve simultaneous improvement of stability and controllable communication request with surroundings, a trade-off issue frequently encountered in traditional coacervate systems. Moreover, the proposed strategy can be expanded to include other moieties as functional fasteners and effectors for the construction of even more complex synthetic membraneless organelles to execute sophisticated tasks.

## Supporting Information Available

Experimental section (Reagents and Experimental Methods); oligonucleotide sequence tables (Tables S1–S3) and supporting figures (Figures S1–S38) (PDF)
