# Supplementary material for: Spatiotemporal Regulation of Cell Fate in Living Systems Using Photoactivatable Artificial DNA Membraneless Organelles
Source: ACS Cent Sci. 2024 May 21;10(6):1201–10. doi: 10.1021/acscentsci.4c00380 (PMC11212128; doi:10.1021/acscentsci.4c00380)
Supplement: Supplementary file 1 — oc4c00380_si_001.pdf [file oc4c00380_si_001.pdf]

Supplementary Materials for

Spatiotemporal Regulation of Cell Fate in Living Systems Using Photoactivatable Artificial DNA Membraneless Organelles

Lili Zhang[1], Mei Chen[1], Zhiqiang Wang[1], Minjuan Zhong[1], Hong Chen[1], Ting Li[1], Linlin Wang[1], Zhihui Zhao[1], Xiao-Bing Zhang[1], Guoliang Ke[1]*, Yanlan Liu[1]*, Weihong Tan[1,2,3]*

[1]*Molecular Science and Biomedicine Laboratory (MBL), State Key Laboratory of Chemo/Biosensing and Chemometrics, College of Chemistry and Chemical Engineering, College of Biology, Aptamer Engineering Center of Hunan Province, Hunan University, Changsha 410082, China*

[2]*The Key Laboratory of Zhejiang Province for Aptamers and Theranostics, Zhejiang Cancer Hospital, Hangzhou Institute of Medicine (HIM), Chinese Academy of Sciences, Hangzhou, Zhejiang 310022, China*

[3]*Institute of Molecular Medicine (IMM), Renji Hospital, Shanghai Jiao Tong University School of Medicine, and College of Chemistry and Chemical Engineering, Shanghai Jiao Tong University, Shanghai 200240, China*

[*] *Corresponding author.*

Email: glke@hnu.edu.cn;

Email: ylliu@hnu.edu.cn;

Email: tan@hnu.edu.cn.

Content

Experimental Section........3

Reagents and characterizations........3

LLPS-driven condensation of metastable long ssDNA membraneless organelles (mLDMO) .3

LLPS-driven condensation of self-stabilized DNA organelles (sLDMO)........4

Stability tests of mLDMO and sLDMO........4

Fastener-bound condensation of DNA MO........5

Agarose gel electrophoresis........5

NIR photoresponsivity of DNA MO........5

Effector loading in photoactivatable DNA MO........6

Photoactive release behaviors........6

Cell lines and cell culture........7

Cell internalization and photoactive drug release studies........7

Cell cytotoxicity........7

Western blot assay........8

Animals........8

Pharmacokinetic and biodistribution studies........8

In vivo spatiotemporally controlled antitumor studies........9

Statistical analysis........10

Tables........11

Figures........12

References........28

Experimental Section

Reagents and characterizations

All DNAs and doxorubicin (Dox) were bought from Sangon Biotech. Co. (Shanghai, China) without further purification. Phi29 DNA polymerase, pyrophosphorylase, and dNTP were obtained from New England Biolabs (Ipswich, MA). E. coli DNA ligase was purchased from Takara Biotech. Co. (Shanghai, China). The predicted secondary structures were shown through the NUPACK program (http://www.nupack.org/partition/histogram_detail/689995?token=gdPZi1G3Yq&strand_id=0).

Fluorescence characterizations were carried out on a FV1000 confocal laser scanning microscope. A JEM-2100 Plus transmission electron microscope and a SUPRA 40 scanning electron microscope were employed for morphology characterization of the products. Element analysis of the samples was performed with inductively coupled plasma optical emission spectroscopy (ICP-OES) and transmission electron microscopy energy-dispersive X-ray spectroscopy (TEM-EDS).

LLPS-driven condensation of metastable long ssDNA membraneless organelles (mLDMO)

The mLDMO was constructed according to a reported reference.[1] The phosphorylated linear template DNA and primer DNA were mixed at 1 μM in 100 μL of 1 x DNA ligation buffer (5 mM Tris-HCl, 1 mM $MgCl_2$, 0.1 mM ATP, and 1 mM DTT). The mixed solution was then heated to 95 °C for 5 min and cooled down to 25 °C at 1 °C/s. After annealing, 10 μL of E. coli DNA Ligase (60 U/μL) were added into the tube, gently mixed, and reacted for 3 h at 16 °C. Thereafter, the enzyme was deactivated through heating the mixture for 20 min at 65 °C.

Next, 20 μL of Exonuclease I (20 U/μL) and 20 μL of Exonuclease III (100 U/μL) were added to the mixture, and the reaction was allowed to proceed overnight at 37 °C, followed by removing the unreacted strands and linear templates. The enzymes were

then deactivated by heating at 80 °C for 30 min. The template DNA was purified by an ultrafiltration device (10 kDa, Merck Millipore) and washed three times with 400 μL of TE buffer. The as-obtained ssDNA was quantified by a Nanodrop 2000 (Fisher Scientific) and diluted to 1 μM with TE buffer.

For RCA, 5 μL of the ligation products (1 μM) were mixed with 2 μL of phi29 DNA polymerase (10 U/μL) in 100 μL of reaction system, which contained 1 x polymerase buffer (50 mM Tris-HCl, 10 mM $(NH_4)_2SO_4$, 4 mM DTT, and 10 mM $MgCl_2$), 1 μL of inorganic pyrophosphatase (2 U/μL), and 5 μL of dNTP (100 mM). The reaction was kept at 30 °C for 60 h before heat-induced deactivation for 10 min at 70 °C. Then, the RCA products were purified by filtration with 30 kDa Millipore three times using 400 μL of TE buffer. The concentration of the long ssDNA was determined by using a Nanodrop 2000.

The mLDMO was prepared by mixing long ssDNA at the concentration of 0.2 g/L in TE buffer with 50 mM $MgAc_2$, followed by heating to 95 °C for 15 min and cooling down to 25 °C at 1 °C/s.

LLPS-driven condensation of self-stabilized DNA organelles (sLDMO)

For RCA, the circular DNA template (0.3 μM) was incubated with phi29 DNA polymerase (2 U/μL), dNTP (100 mM/μL), and BSA (1 x) in 1 x polymerase buffer (50 mM Tris-HCl, 10 mM $(NH_4)_2SO_4$, 10 mM $MgCl_2$, and 4 mM DTT) at 30 °C.[2] The RCA reaction was allowed to proceed for indicated time intervals (24 h, 48 h, and 60 h) and terminated by heating at 95 °C for 10 min. The as-formed sLDMO were washed with double-distilled H_2O (ddH_2O), precipitated by centrifugation, and qualified using the Nanodrop. Finally, the sLDMO was diluted to 0.2 g/L for later use.

The scattering of long ssDNA, mLDMO and sLDMO was first detected by a UV2450 ultraviolet spectrophotometer (Shimadzu Co., Kyoto, Japan).

Stability tests of mLDMO and sLDMO

To study the physiological stability of mLDMO and sLDMO, 2 μL of Cy5-DNA/FAM-

DNA (100 μM) were added to 10 μL of mLDMO/sLDMO solution (0.2 g/L) for 1 h at room temperature for hybridization to form mLDMO/Cy5, mLDMO/FAM, sLDMO/Cy5, or sLDMO/FAM before CLSM. 10 μL of these products were suspended in 100 μL of PBS for imaging with a FV1000 confocal laser scanning microscope (CLSM).

Fastener-bound condensation of DNA MO

To endow DNA MO with spatiotemporally controlled activity, NIR absorbing Pd NPs were prepared according to a previous method and used as the photoresponsive fastener.[3] Briefly, 50 μL of Pd NPs (0.5 mg/mL) were first washed with 500 μL acetone. After centrifugation at 7000 rpm for 2 min, Pd NPs were redispersed in 10 μL fresh deionized water. 5 μL of Pd NPs aqueous solution were mixed with 5 μL of mLDMO or sLDMO for 48 h to form photoactivatable coacervates (pmLDMO or psLDMO).

To investigate the stability of pmLDMO and psLDMO, they were labeled with Cy5 under the same procedures as noted above. The resulting pmLDMO/Cy5 or psLDMO/Cy5 was dispersed in 100 μL of PBS and imaged at the indicated incubation time (0, 0.5 h, 2 h, 4 h, 12 h, and 24 h) by CLSM.

Agarose gel electrophoresis

The stepwise formation of long ssDNA, mLDMO, and sLDMO was evaluated by 2% agarose gel electrophoresis for 30 min (120 V). After staining with Gel Red, the gel was imaged with a Gel Doc XR system (Bio-Rad).

NIR photoresponsivity of DNA MO

To study the NIR photoresponsive activity of DNA MO, FAM and Cy5 were loaded in psLDMO, and the NIR photoresponsivity of these MO was studied by fluorescence imaging. Briefly, 4 μL of FAM-DNA (100 μM) were mixed with 20 μL of psLDMO, followed by incubation with 20 μL of Pd NPs aqueous solution for 48 h. After washing with PBS three times, 4 μL of the Cy5-NH_2 solution (1 mg/mL) and the resulting

psLDMO/FAM were mixed in 100 μL of PBS for 24 h at 37 °C to yield psLDMO/FAM/Cy5. Thereafter, 200 μL of the resulting psLDMO/FAM/Cy5 (0.02 μM) were exposed to an 808 nm laser (100% of the maximum intensity ≤ 10 W/cm^2) for 1 s.[1] After centrifugation, fluorescence of the supernatant was measured to determine the release of Cy5 from psLDMO/FAM/Cy5, while the pellet was imaged by CLSM. This process was repeated five times. As a negative control, Cy5 release from Pd NPs-free sLDMO/FAM/Cy5 was also studied.

Effector loading in photoactivatable DNA MO

As a proof-of-concept study, Dox was used as the model effector molecules and loaded within the photoactivatable DNA MO. To do this, 20 μL of psLDMO (0.3 μM) were mixed with 10 μM Dox dispersed in 100 μL of PBS for 24 h. Afterwards, the mixture was centrifuged and washed at 13000 rpm for 5 min. The supernatants were collected, and the amount of Dox in the supernatant was quantified by measuring fluorescence (Ex: 488 nm; Em: 590 nm). The loading efficiency of Dox in DNA MO was calculated as loading Dox = Total Dox - Dox in supernatant.

Photoactive release behaviors

To investigate the spatiotemporally controlled activity of the engineered DNA MO, their photothermal effects in response to NIR light were examined. Briefly, 100 μL of psLDMO aqueous solutions (0.005 μM, 0.01 μM, and 0.02 μM) were irradiated by an 808 nm NIR laser (2.0 W/cm^2) for 5 min, and the solution temperature was recorded with an IR thermal imaging system. Thereafter, 100 μL of psLDMO aqueous suspension (0.02 μM) were irradiated by an 808 nm laser at 2.0 W/cm^2 for 5 min, and the power was switched off. Such ON/OFF irradiation process was repeated five times, and the temperature changes of the psLDMO aqueous suspension in each cycle was determined in order to evaluate the photostability of psLDMO.

Next, the photoactive release dynamics of Dox from psLDMO/Dox was studied. 200 μL of psLDMO/Dox (0.02 μM) were irradiated by an 808 nm NIR laser at a 2.0

W/cm^2 power density for 5 min. The solution was centrifuged at 13000 rpm for 5 min, and the released Dox was quantified by measuring the fluorescence of Dox in the supernatant as

Dox release percent (%) = released Dox/Dox encapsulated into psLDMO * 100%

The above experiment was repeated 5 times, and the total released Dox in each cycle was calculated. As a negative control, the release of Dox from psLDMO/Dox in the dark within the same time period was also determined.

Cell lines and cell culture

4T1 cancer cells were obtained from the American Type Culture Collection (Manassas, VA) and cultured in 1640 medium with 10% fetal bovine serum (FBS) (Zeta) and 1% penicillin-streptomycin (GIBCO) at 37 °C in a humid atmosphere with 5% CO_2.

Cell internalization and photoactive drug release studies

To study cell internalization of these DNA MO, 10^5 4T1 cells per dish were seeded in confocal dishes and cultured for 24 h. The medium was removed, and a fresh medium containing psLDMO/Dox (5 μM equivalent of Dox) was added. Cells were then incubated for an additional 24 h. After washing with PBS twice, cells were irradiated by an 808 nm laser at 2.0 W/cm^2 for 5 min and cultured in fresh DMEM medium for 2 h. Cell internalization and Dox release were evaluated by CLSM.

Cell cytotoxicity

To evaluate the *in vitro* spatiotemporally controlled antitumor effect of these photoactivatable DNA coacervates, 4 T1 cells were seeded in wells of a 96-well plate (5×10^3 cells per well) and cultured for 24 h. Cells were then treated with psLDMO, psLDMO/Dox, and free Dox, respectively. The dosage of Dox was maintained at 5 μM, and the concentration of psLDMO was kept the same as that for the psLDMO and psLDMO/Dox groups. After 24-h incubation, cells were washed with PBS twice. Thereafter, two groups of cells treated with psLDMO or psLDMO/Dox were irradiated

by an 808 nm laser at 2.0 W/cm^2 for 5 min. After culture for an additional 24 h, cell viability of these groups was determined by the CCK-8 assay.

Western blot assay

4T1 cells treated with psLDMO or psLDMO/Dox in the absence or presence of laser irradiation were harvested and lysed with a lysis buffer (50 mM Tris-HCl pH 7.4, 150 mM NaCl, 1% NP-40 substitute, 0.25% sodium deoxycholate, 1 mM sodium fluoride, 1 mM Na_3VO_4, and 1 mM EDTA), which was supplemented with protease inhibitor cocktail (Cell Signaling) and 1 mM phenylmethanesulfonyl fluoride. The protein concentration in each group was determined by a bicinchoninic acid (BCA) protein assay kit (Pierce/Thermo Scientific). Afterwards, 30 μg protein from each group were loaded on the SDS-PAGE gel and transferred to a PVDF membrane. The gel was then blocked with 5% skim milk in TBST (50 mM Tris-HCl, pH 7.4, 150 mM NaCl, and 0.1% Tween 20) and kept in a primary antibody solution at 4 ºC overnight.

After washing three times, the membrane was incubated in HRP-conjugated secondary antibody solution at room temperature for 1 h. Expression of the targeted protein was investigated by the enhanced chemiluminescence (ECL) detection system.

Animals

BALB/c mice were purchased from Hunan SJA Laboratory Animal Co. Ltd. Animal care and handling procedures were performed in accordance with the guidelines of the Institutional Animal Care and Use Committee of Hunan University and the guidelines of the Regional Ethics Committee for Animal Experiments. The animal experiments invovled in this work have all been approved by the Animal Care and Use Committee of Hunan University (SYXK 2018-0006).

Pharmacokinetic and biodistribution studies

Cy5-lableld psLDMO was prepared for pharmacokinetic and biodistribution studies. For pharmacokinetic study, six-week-old BALB/c mice (n = 3) were injected with

psLDMO/Cy5 through the tail vein at a dose of 2.0 nmol Cy5-DNA per mouse. At the indicated time points (0.15, 0.5, 1, 2, 4, 8, and 12 h), 20 μL of blood were collected from the retroorbital plexus of mouse eye and diluted to 100 μL with PBS containing 1 mg/mL heparin sulfate. The fluorescence intensity of blood was measured by using a Tecan spark-multimode microplate reader.

For biodistribution study, 4T1 tumor-bearing BALB/c mice were established by subcutaneous implantation of 10^6 4T1 cells in PBS on the backside of six-week-old mice. Next, 200 μL of psLDMO/Cy5 in PBS were intravenously administered into 4T1 tumor-bearing BALB/c mice ($n = 3$) via the tail vein at 2.0 nmol Cy5-DNA per mouse. At the indicated time intervals, mice were anesthetized with 2.5% isoflurane and imaged with an IVIS Lumina II imaging system (Caliper Life Science, USA). At 24 h post-injection, mice were sacrificed, and both tumor tissues and main organs (heart, liver, spleen, lung, and kidney) were collected for imaging.

In vivo **spatiotemporally controlled antitumor studies**

4T1 xenograft tumor-bearing mice were randomly divided into seven groups when the tumor size reached ~ 100 mm^3 and subjected to the following treatments: (i) PBS, (ii) PBS + laser, (iii) psLDMO, (iv) psLDMO + laser, (v), free Dox, (vi) psLDMO/Dox, and (vii) psLDMO/Dox + laser. All samples were injected through the tail vein. For groups (v) to (vii), the dosage of Dox was maintained at 5 mg/kg, and the concentration of psLDMO in groups (iii) and (vi) was kept the same as that for groups (v) to (vii). At 24 h post-injection, the mice in groups (ii), (iv) and (vii) were irradiated with an 808 nm laser with power density at 2 W/cm^2 for 5 min. Tumor temperature in each group was monitored. Afterwards, tumor size and body weight were monitored every day. Tumor volume was calculated as Volume = length x $width^2/2$. The relative volume of tumors was defined by normalizing the measured values to their initial sizes. Mice were finally sacrificed for histopathological analysis, including H&E, immunohistochemical, and immunofluorescence staining.

Statistical analysis

Data were demonstrated as their means with standard deviation (SD), unless otherwise noted. Statistical analysis of data was conducted by Student's t-test.

Tables

Table S1. DNA sequences for RCA.

Name	Sequences (5'-3')
Template	/5Phos/ATCTATCCTAATTTTTTTTTTTTTTTTTTTTGAACCCGTAT
Primer	TTAGGATAGATATACGGGTTC
Cy5-DNA	/Cy5/TGAACCCGTATATCTATCCTAA
FAM-DNA	/6-FAM/TGAACCCGTATATCTATCCTAA

Table S2. ICP-OES analysis of palladium in sLDMO and psLDMO.

Sample	Concentration (mg/L)	Standard deviation (SD)
sLDMO	-0.028	0.001
psLDMO	0.498	0.009

Table S3. ICP-OES analysis of intracellular palladium after incubation with or without psLDMO

Sample	Concentration (mg/L)	Standard deviation (SD)
Cell	-0.012	0.001
Cell incubated with psLDMO	0.132	0.002

Figures

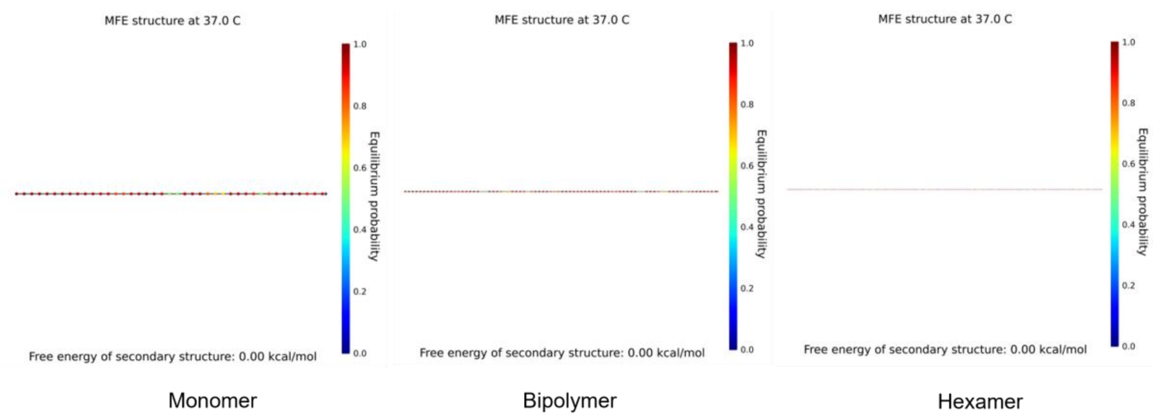

Figure S1. Predicted secondary structures of the sequences used for RCA.

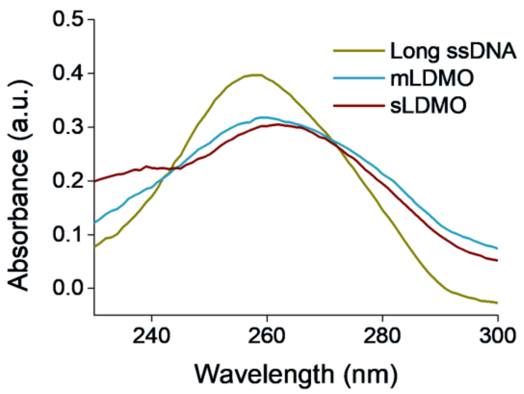

Figure S2. UV-vis spectra of long ssDNA, the metastable DNA coacervates (mLDMO) and stable DNA coacervates (sLDMO).

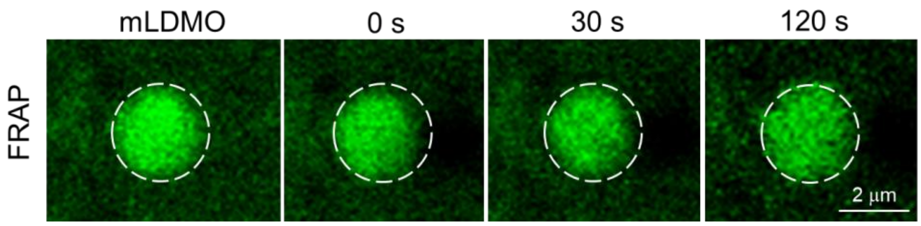

Figure S3. Confocal imaging of FAM-labeled mLDMO for fluorescence recovery after photobleaching analysis.

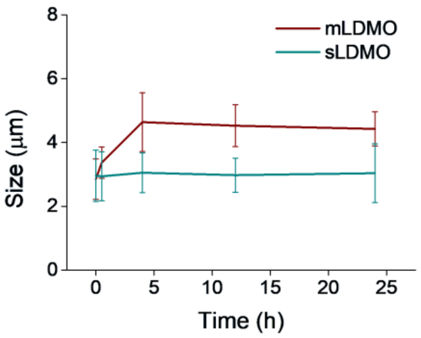

Figure S4. Quantitative analysis of the size of coacervates in Figure 2e.

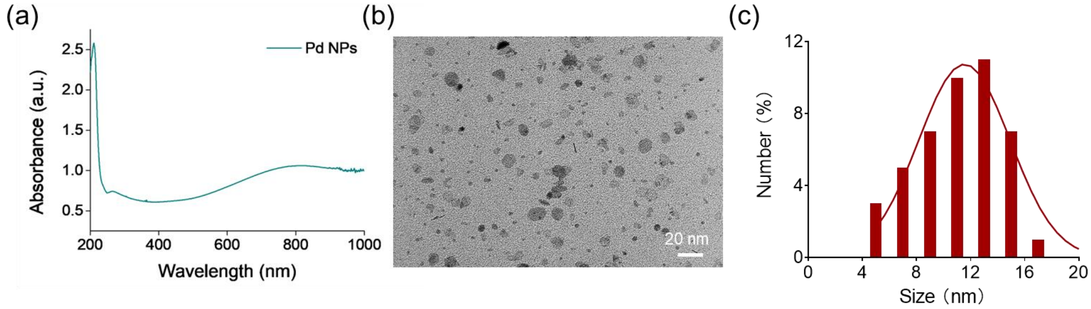

Figure S5. (a) UV-vis spectrum and (b) TEM imagin of Pd NPs. (c) Size distribution statistics as calculated from panel b.

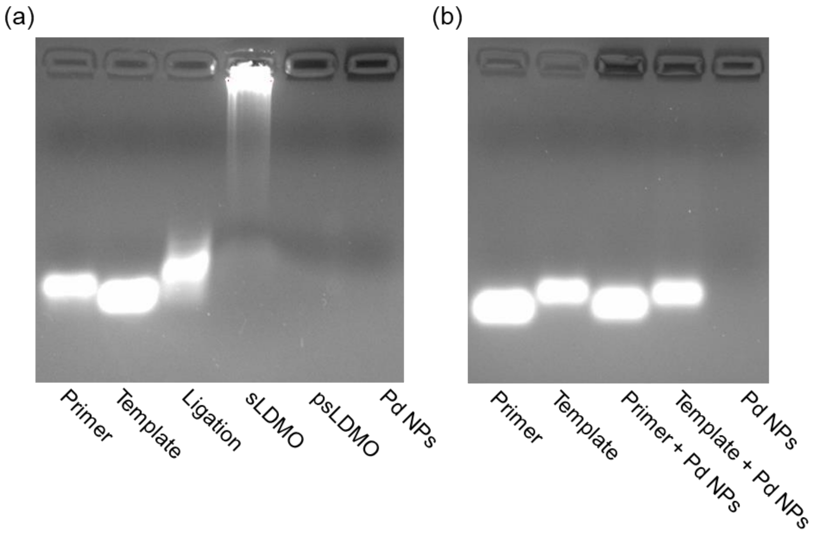

Figure S6. (a) Gel electrophoresis analysis of the stepwise formation of sLDMO with *vs.* without binding with Pd NPs. (b) Gel electrophoresis of the used primer and template DNA sequences in the absence *vs.* presence of Pd NPs.

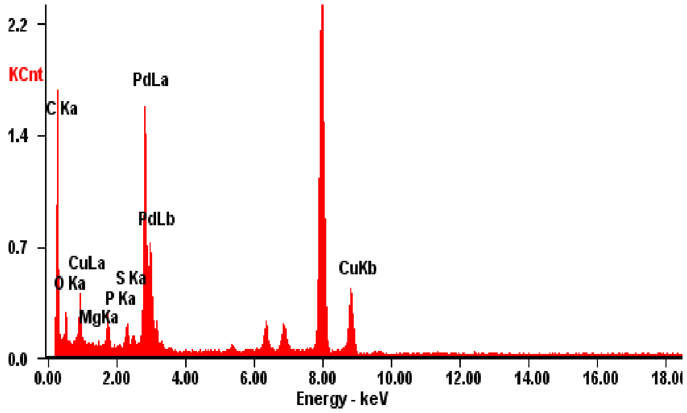

Figure S7. TEM-EDS analysis of psLDMO.

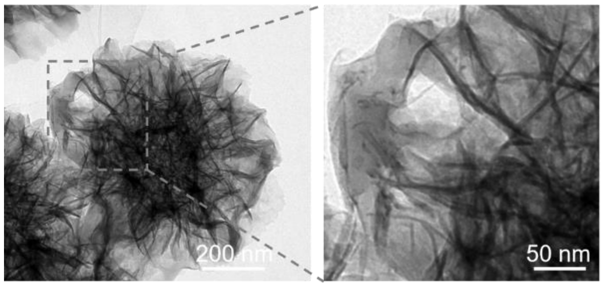

Figure S8. TEM imaging and magnified TEM imaging of sLDMO after incubation with a low concentration of Pd NPs (0.125 ng/mL).

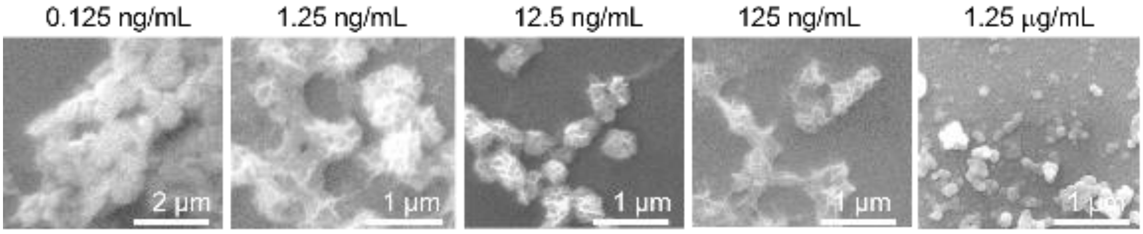

Figure S9. SEM imaging of sLDMO after incubation with Pd NPs at different concentrations.

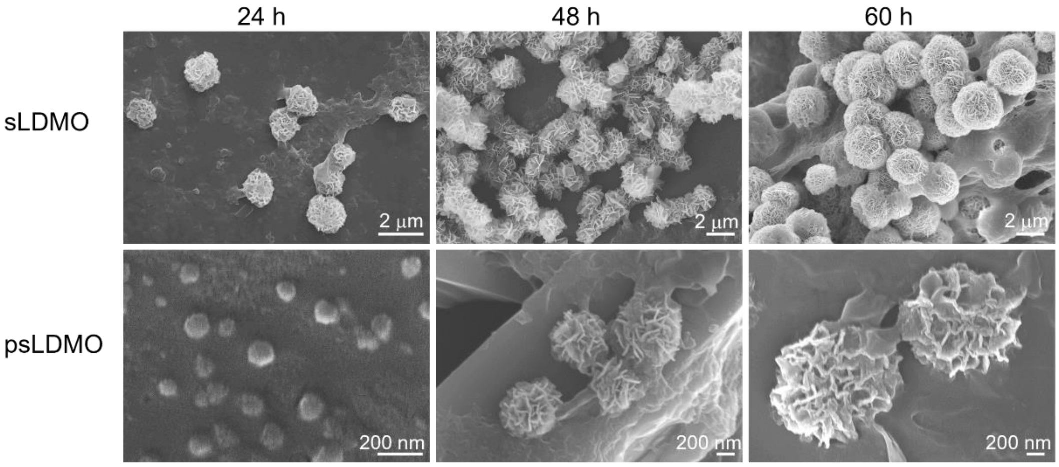

Figure S10. SEM imaging of LLPS-driven formation of sLDMO with different RCA reaction durations, as well as corresponding fastener-bound condensation of sLDMO with Pd NPs.

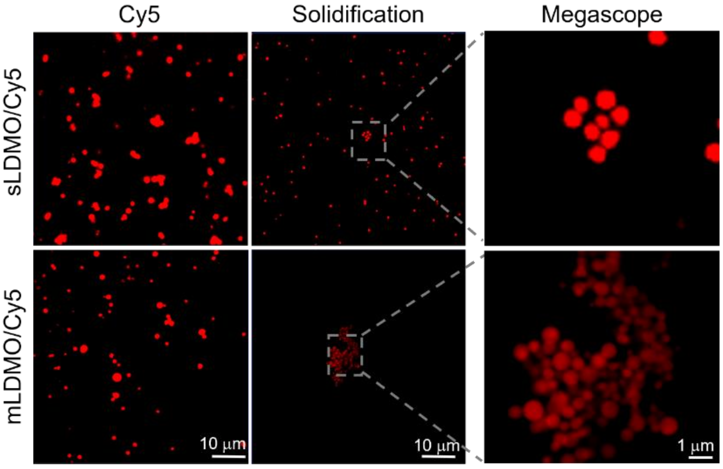

Figure S11. Representative fluorescent images of mLDMO/Cy5 and sLDMO/Cy5 before *vs.* after condensation with Pd NPs.

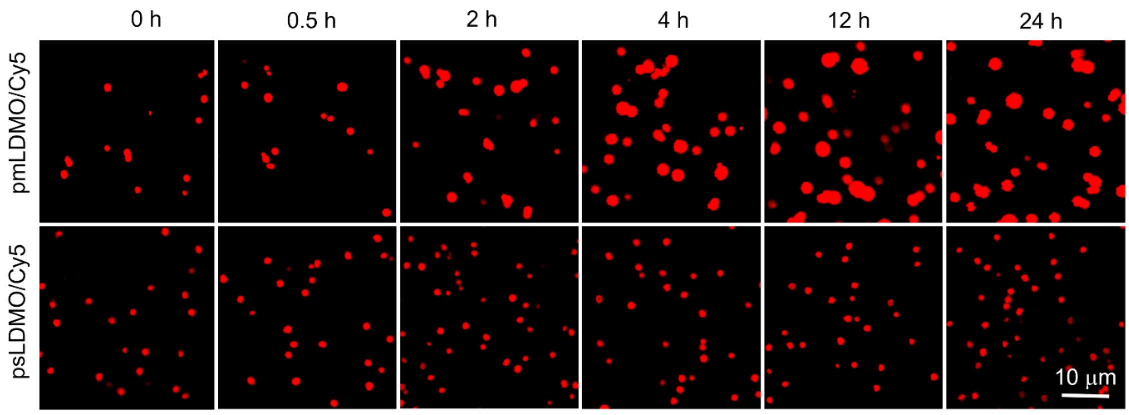

Figure S12. Confocal imaging of Cy5-labeled pmLDMO and psLDMO after incubation in hypotonic PBS buffer for different time intervals.

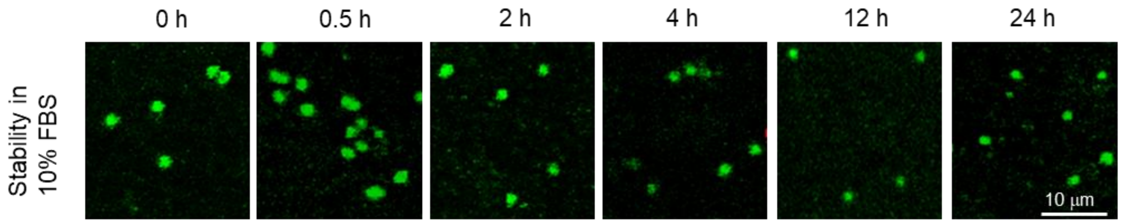

Figure S13. Confocal imaging of FAM-labeled psLDMO after incubation in 10% FBS buffer for different time intervals.

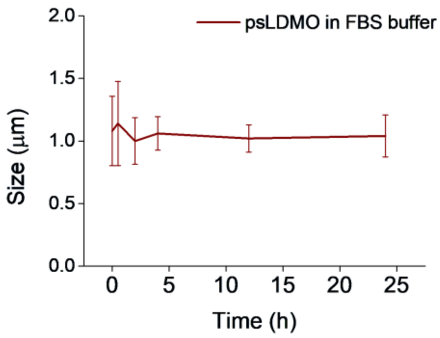

Figure S14. Quantitative analysis of confocal images in Figure S13.

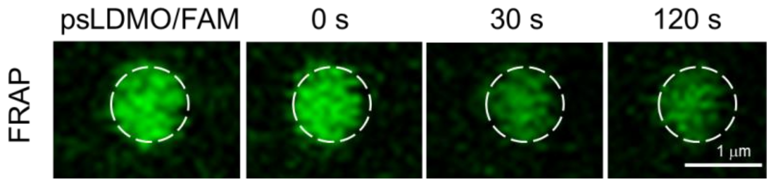

Figure S15. Confocal imaging of FAM-labeled psLDMO for fluorescence recovery after photobleaching analysis.

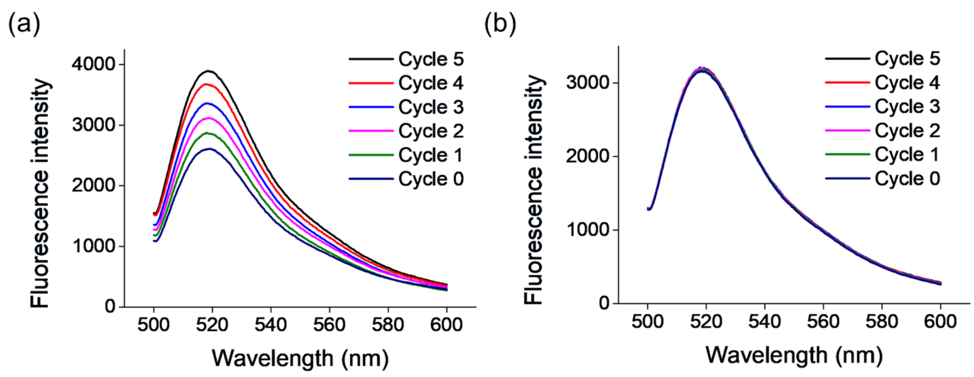

Figure S16. Fluorescence spectra of FAM release from FAM-labeled psLDMO (a) and FAM-labeled sLDMO (b) during five cycles of laser irradiation (808 nm, 10 W/cm^2, 1 s).

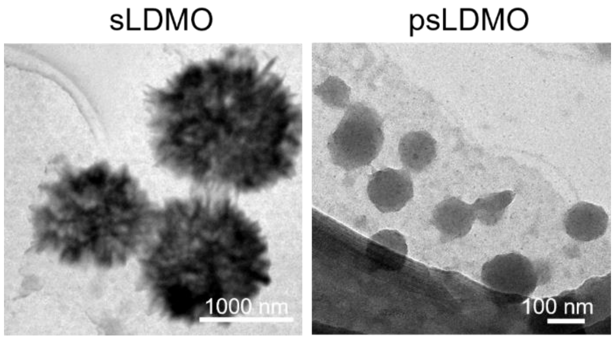

Figure S17. TEM imaging of psLDMO obtained after 24-h RCA before and after binding with Pd NPs.

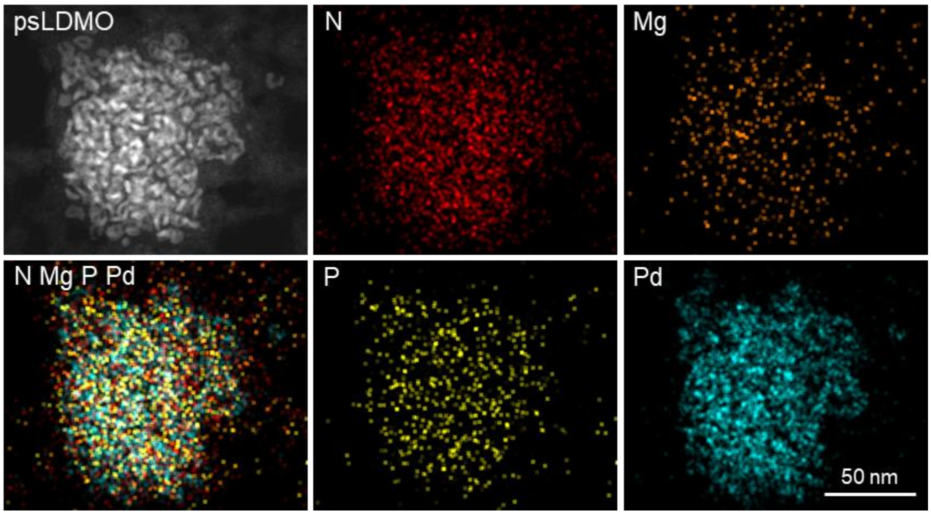

Figure S18. STEM-based EDS mapping of psLDMO.

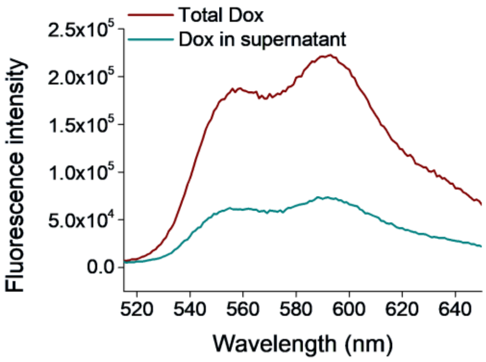

Figure S19. Fluorescence spectra of Dox loaded into psLDMO.

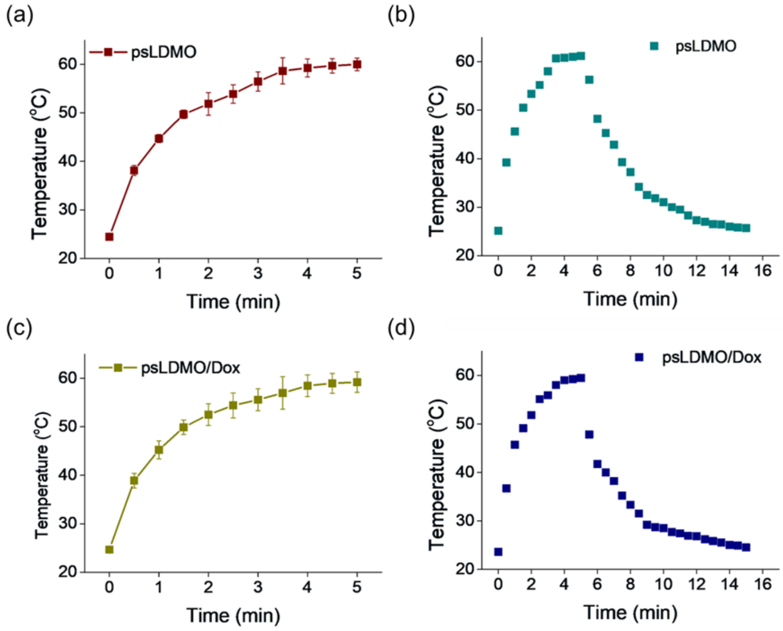

Figure S20. Time-dependent temperature changes (a, c) and Heating/cooling profile (b, d) of psLDMO and psLDMO/Dox aqueous solutions (0.02 μM) under laser irradiation (808 nm, 2 W/cm^2).

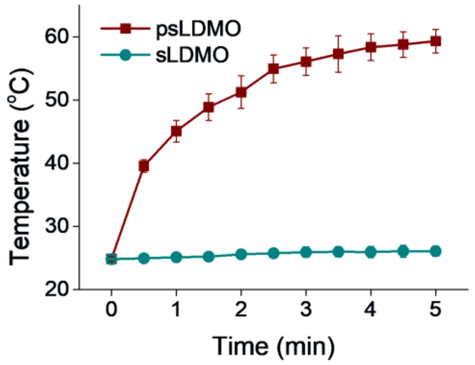

Figure S21. Time-dependent temperature changes of psLDMO and sLDMO aqueous solutions (0.02 μM) under laser irradiation (808 nm, 2 W/cm^2)

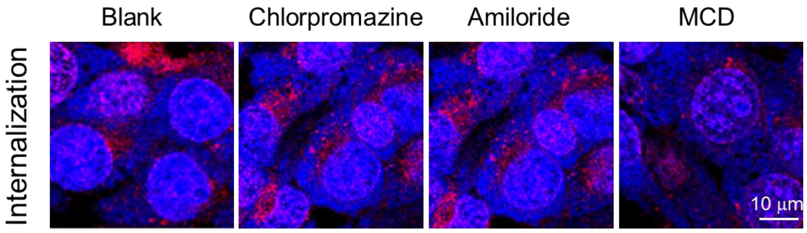

Figure S22. Confocal imaging of psLDMO/Dox-treated 4T1 cells after preincubation with methyl-ß-cyclodextrin (MCD), chlorpromazine, and amiloride.

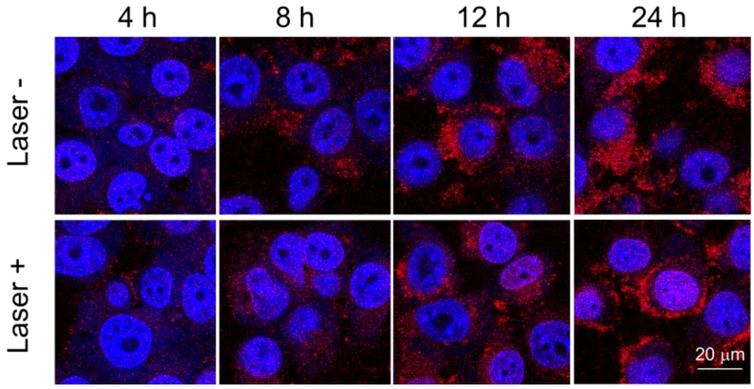

Figure S23. Confocal imaging of psLDMO/Dox-incubated 4T1 cells with vs. without 5-min laser irradiation after incubation for 4, 8, 12, and 24 h, respectively.

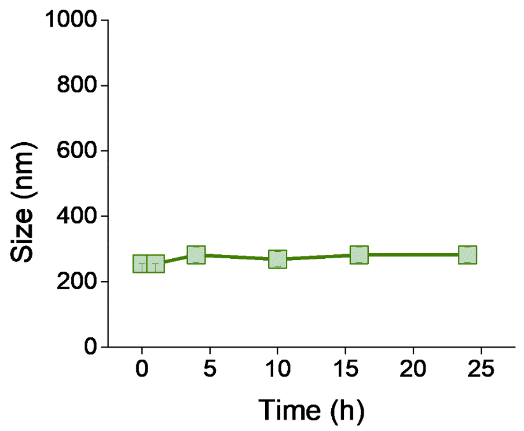

Figure S24. DLS analysis psLDMO size changes in 10% FBS.

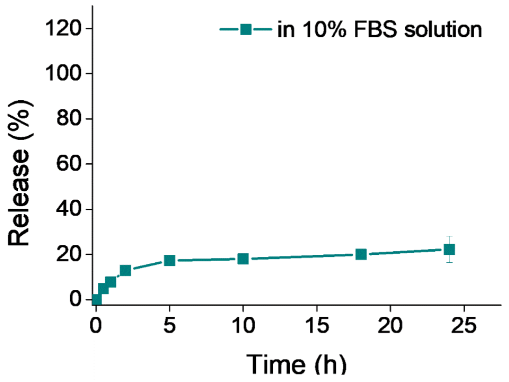

Figure S25. Time-dependent release of Dox from psLDMO/Dox suspended in the 10% FBS.

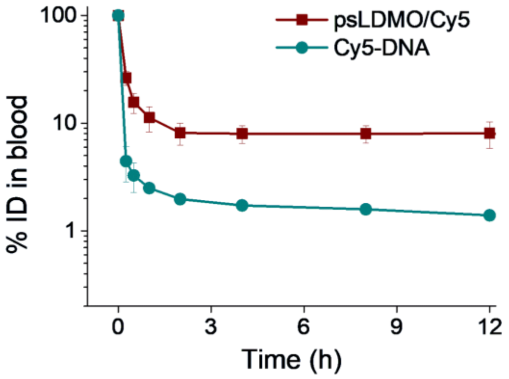

Figure S26. Pharmacokinetics of psLDMO/Cy5 and free Cy5-DNA in blood upon intravenous injection.

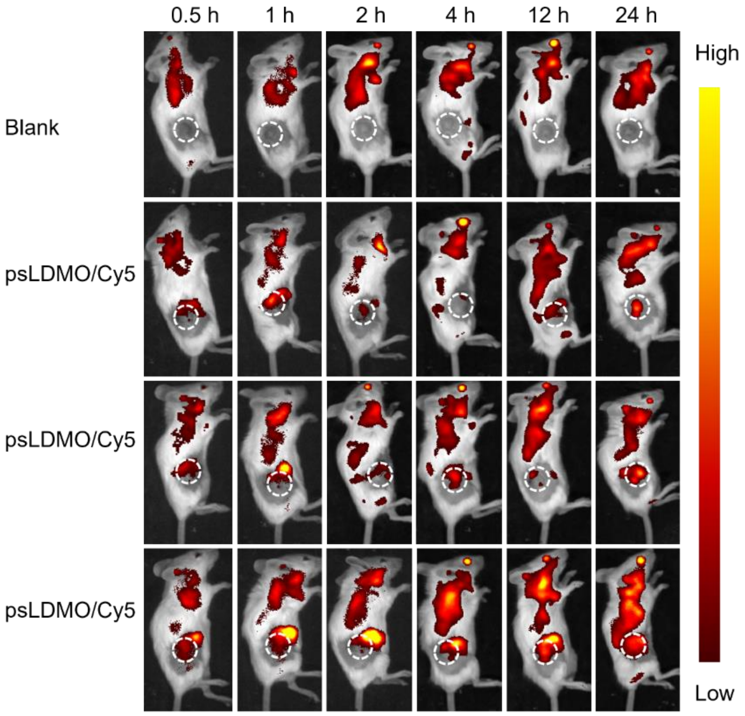

Figure S27. Time-dependent whole-body fluorescence imaging of 4T1 tumor-bearing mice after intravenous injection of psLDMO/Cy5.

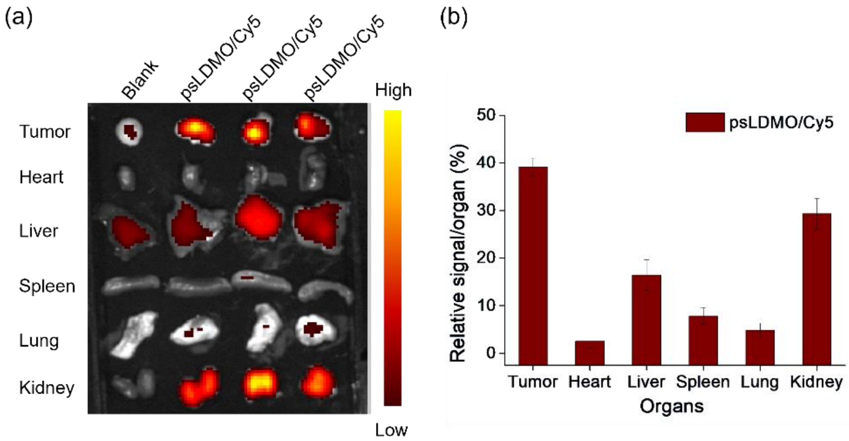

Figure S28. (a) Fluorescence imaging of tumor tissues and main organs harvested from psLDMO/Cy5-treated mice 24-h post-intravenous injection. (b) Quantitative determination of biodistribution of psLDMO/Cy5 in 4T1 tumor-bearing mice.

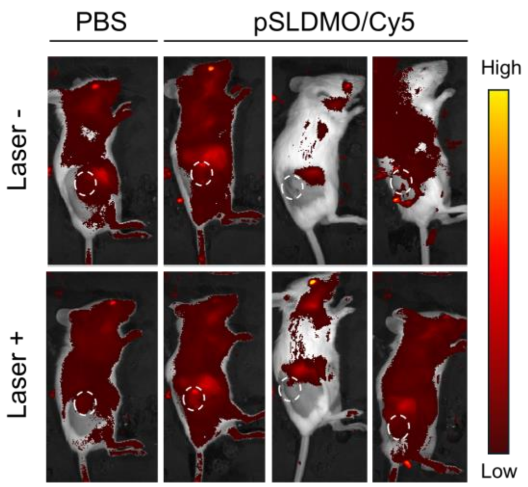

Figure S29. Whole-body fluorescence imaging of 4T1 tumor-bearing mice with vs. without 5-min laser irradiation after intravenous injection of psLDMO/Cy5.

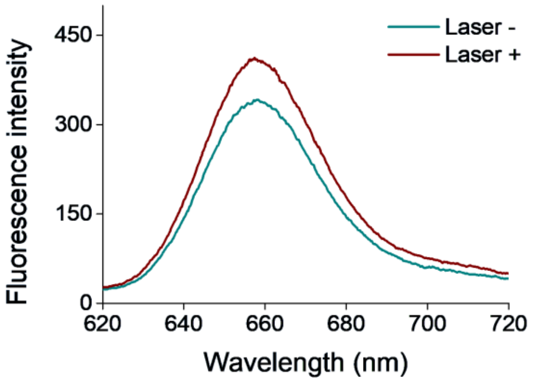

Figure S30. Fluorescence spectra of psLDMO/Cy5 with vs. without 5-min laser irradiation (2 W/cm^2, 5 min).

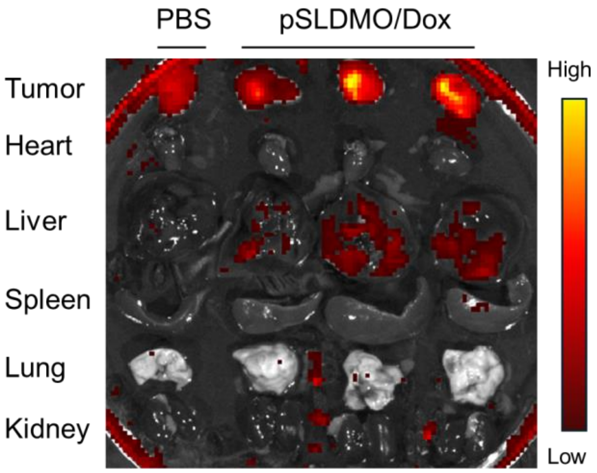

Figure S31. Fluorescence imaging of tumor tissues and main organs harvested from psLDMO/Dox-treated mice at 24 h post injection.

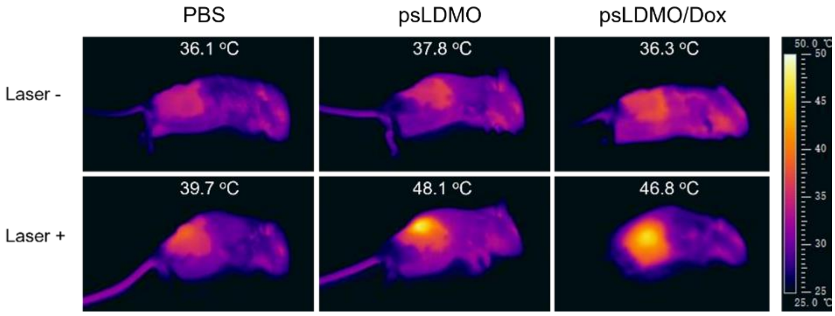

Figure S32. Representative IR images of 4T1 tumor-bearing mice irradiated by an 808 nm laser (2 W/cm^2, 5 min) 24-h post-intravenous injection of PBS, psLDMO, or psLDMO/Dox.

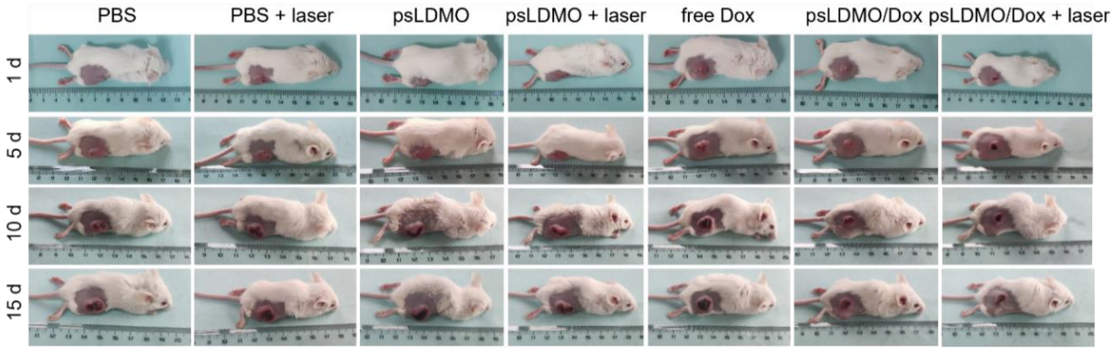

Figure S33. Digital pictures of 4T1 tumor-bearing mice on different days after treatment with PBS, PBS + laser, psLDMO, psLDMO + laser, free Dox, psLDMO/Dox, and psLDMO/Dox + laser, respectively.

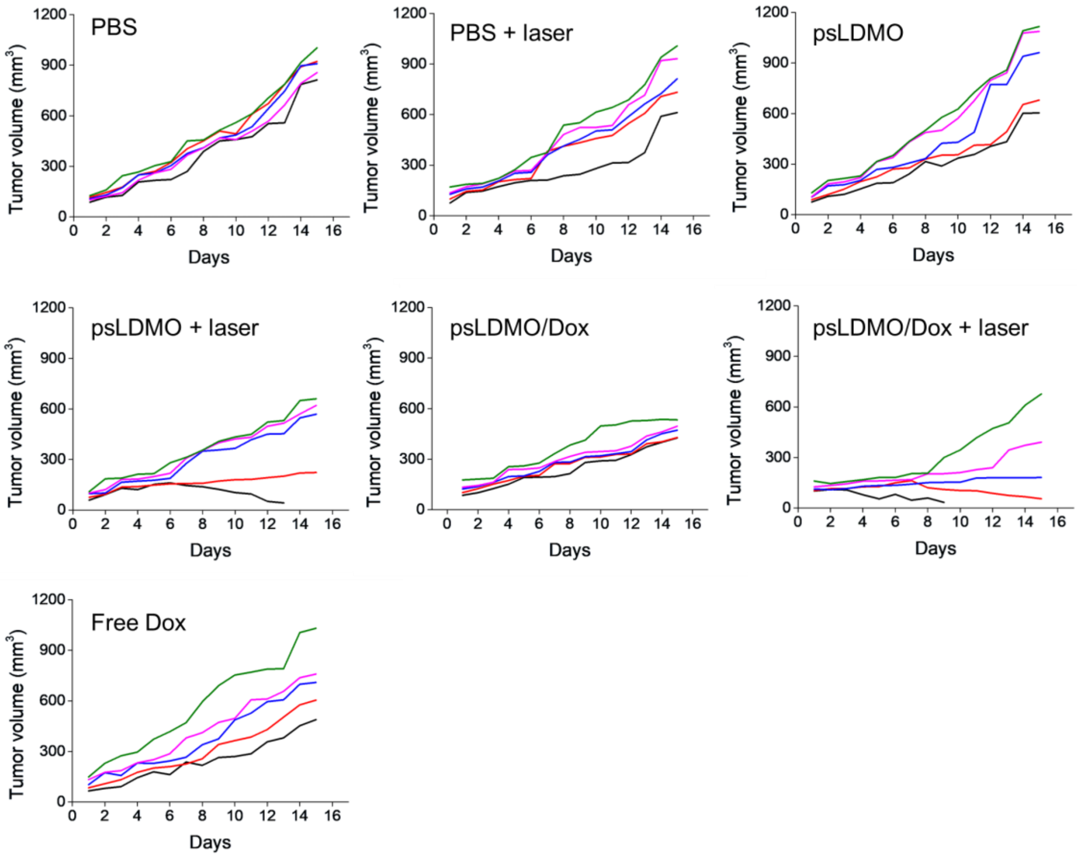

Figure S34. Individual tumor growth curves of 4T1 tumor-bearing mice after different treatments.

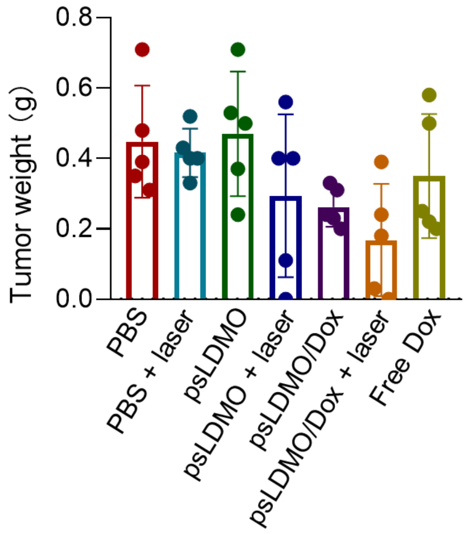

Figure S35. Tumor weights of 4T1 tumor-bearing mice harvested from different treatment groups. Data were presented as mean values ± S.D.

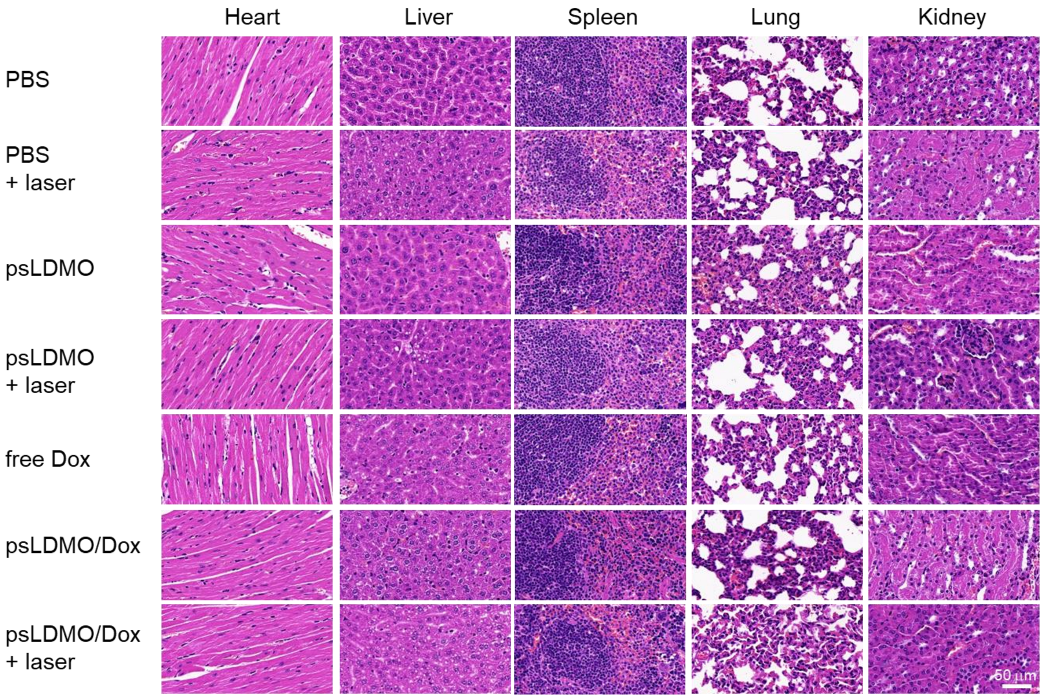

Figure S36. Representative H&E staining images of tissue sections harvested from 4T1 tumor-bearing mice on day 15 after treatment with PBS, PBS + laser, psLDMO, psLDMO + laser, free Dox, psLDMO/Dox, and psLDMO/Dox + laser, respectively.

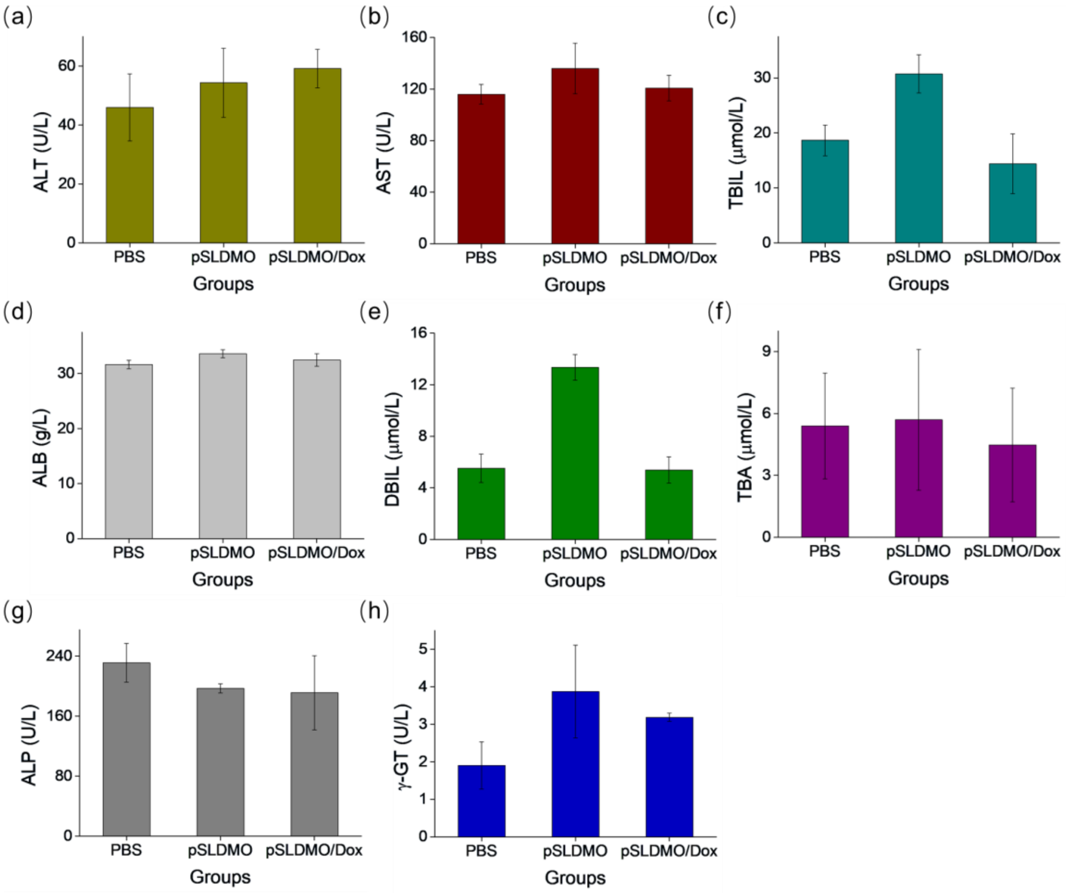

Figure S37. Analysis of blood biochemical indicators in BALB/c mice after treatment with PBS and psLDMO and psLDMO/Dox, including ALT (a), AST (b), TBIL (c), ALB (d), DBIL (e), TBA (f), ALP (g), r-GT (h). Data were presented as mean values ± S.D. The normal ranges of these liver function indicators are 10.06-96.47 U/L (ALT), 36.31-235.48 U/L (AST), 6.09-53.06 μmol/L (TBIL), 21.22-39.15 g/L (ALB), 0.45-33.89 μmol/L (TBIL), 0-8.51 μmol/L (TBA), 22.52-474.35 U/L (ALP), 0-7.78 U/L (γ-GT).[4] ALT: alanine transaminase, AST: aspertate aminotransferase, TBIL: total bilirubin, ALB: albumin, DBIL: direct bilirubin, TBA: total bile acid, ALP: alkaline phosphatase, γ-GT: γ-glutamyltransferase.

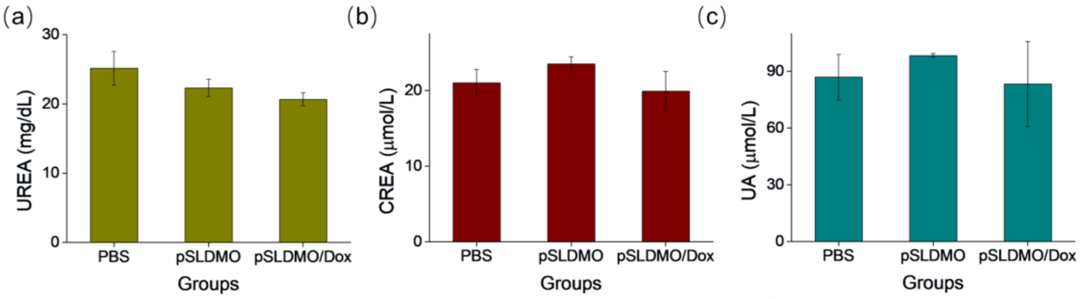

Figure S38. Analysis of blood biochemical indicators in BALB/c mice after treatment with PBS and psLDMO and psLDMO/Dox, including UREA (a), CREA (b), UA (c). Data were presented as mean values ± S.D. The normal ranges of these kidney function indicators are 10.81-34.74 mg/dL (UREA), 10.91-85.09 μmol/L (CREA), and 44.42-224.77 μmol/L (UA).[4] CREA: creatinine, UA: uric acid.

References

[1] Merindol, R.; Loescher, S.; Samanta, A.; Walther, A. Pathway-controlled formation of mesostructured all-DNA colloids and superstructures. *Nat. Nanotech.* **2018**, *13* (8), 730-738.

[2] Zhang, L.; Wang, L.; Yuan, X.; Zhong, M.; Chen, H.; Zhang, D.; Han, X.; Xie, S.; He, L.; Li, Y.; Chen, F.; Liu, Y.; Tan, W. Decoding the complex free radical cascade by using a DNA framework-based artificial DNA encoder. *Angew. Chem. Int. Ed.* **2021**, *60* (19), 10745-10755.

[3] Chen, M.; Chen, S.; He, C.; Mo, S.; Wang, X.; Liu, G.; Zheng, N. Safety profile of two-dimensional Pd nanosheets for photothermal therapy and photoacoustic imaging. *Nano Res.* **2017**, *10* (4), 1234-1248.

[4] Xu, X.; Li, S.; Yu, W.; Yao, S.; Fan, H.; Guo, Z. Activation of RIG-I/MDA5 signaling and inhibition of CD47-SIRPα checkpoint with a dual siRNA-assembled nanoadjuvant for robust cancer immunotherapy. *Angew. Chem. Int. Ed.* **2024**, *136* (10), e202318544.
